# Oligosaccharides and Complex Carbohydrates: A New Paradigm for Cranberry Bioactivity

**DOI:** 10.3390/molecules25040881

**Published:** 2020-02-17

**Authors:** Christina M. Coleman, Daneel Ferreira

**Affiliations:** Department of BioMolecular Sciences, Division of Pharmacognosy, and the Research Institute of Pharmaceutical Sciences, School of Pharmacy, University of Mississippi, University, MS 38677, USA

**Keywords:** xyloglucan, prebiotic, diet-microbiota interaction, UTI, ELSD, *Vaccinium*

## Abstract

Cranberry is a well-known functional food, but the compounds directly responsible for many of its reported health benefits remain unidentified. Complex carbohydrates, specifically xyloglucan and pectic oligosaccharides, are the newest recognized class of biologically active compounds identified in cranberry materials. Cranberry oligosaccharides have shown similar biological properties as other dietary oligosaccharides, including effects on bacterial adhesion, biofilm formation, and microbial growth. Immunomodulatory and anti-inflammatory activity has also been observed. Oligosaccharides may therefore be significant contributors to many of the health benefits associated with cranberry products. Soluble oligosaccharides are present at relatively high concentrations (~20% *w*/*w* or greater) in many cranberry materials, and yet their possible contributions to biological activity have remained unrecognized. This is partly due to the inherent difficulty of detecting these compounds without intentionally seeking them. Inconsistencies in product descriptions and terminology have led to additional confusion regarding cranberry product composition and the possible presence of oligosaccharides. This review will present our current understanding of cranberry oligosaccharides and will discuss their occurrence, structures, ADME, biological properties, and possible prebiotic effects for both gut and urinary tract microbiota. Our hope is that future investigators will consider these compounds as possible significant contributors to the observed biological effects of cranberry.

## 1. Cranberry as a Functional Food

A broad diversity of health benefits has been attributed to the fruit of the American cranberry (*Vaccinium macrocarpon*, Ait. Ericaceae), and numerous detailed reviews have discussed the chemistry and biological properties of previously identified compounds from various cranberry materials [1,2,3]. Some of the reported health benefits of cranberry include the prevention of urinary tract infections (UTIs) [4,5,6], the promotion of overall gut health [7], the promotion of oral health [8,9], and the prevention of gum disease [10,11]. Clinical studies have also investigated cranberry for its ability to help with chronic conditions such as diabetes, high cholesterol, cardiovascular diseases, and even cancer [7,12,13,14,15,16,17].

Perhaps the best known benefit of cranberry consumption is the promotion of urinary tract (UT) health, but with each new systematic review of the clinical literature the recommendation seems to change regarding whether the use of cranberry products for the prevention of UTIs is scientifically supported [18,19,20,21,22,23]. Much of this controversy may arise from limited characterization of the cranberry materials used, a lack of understanding of the cranberry constituents that directly impact UT health, and a lack of awareness of the role of the UT microbiota in overall health and disease prevention.

**Figure 1:**
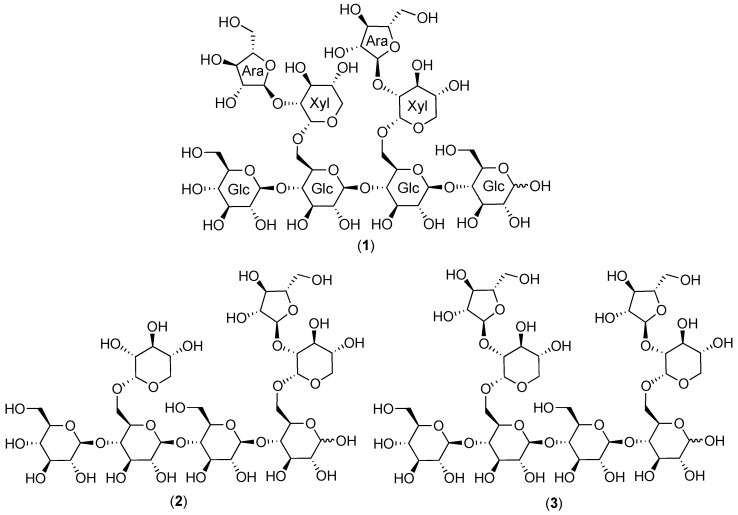
Arabinoxyloglucan GSSG octasaccharide (**1**) isolated from a fraction of porcine urine with bacterial anti-adhesion properties [24,25,26] and arabinoxyloglucan GXGS heptasaccharide (**2**) and GSGS octasaccharide (**3**) isolated from WBP and CHEP cranberry materials [27,28].

**Figure 2:**
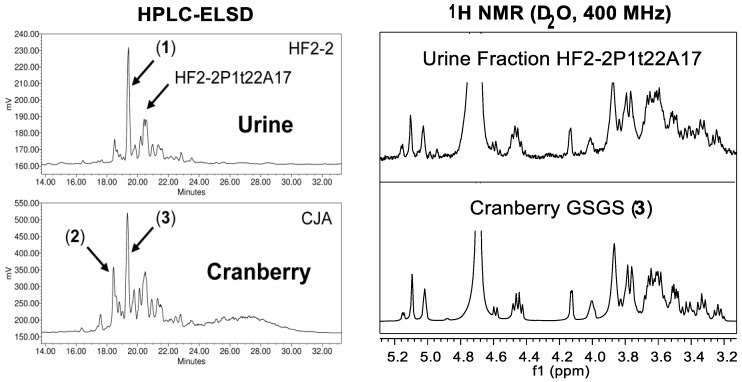
Comparison of HPLC-ELSD elution profiles for oligosaccharide fractions from porcine urine (HF2-2) and the CHEP cranberry material (CJA) that exhibited anti-adhesion properties [26] with a comparison of the ^1^H-NMR spectra (400 MHz, D_2_O) for the purified cranberry GSGS octasaccharide **3** and a purified constituent from porcine urine (HF2-2P1t22A17) that could not be fully elucidated due to quantity limitations [26,28]. Arrows in the HPLC-ELSD chromatograms indicate elution peaks that contained (in addition to other compounds) the urinary GSSG octasaccharide **1**, the cranberry GXGS heptasaccharide **2**, the cranberry GSGS octasaccharide **3** and the urinary oligosaccharide HF2-2P1t22A17.

The newest recognized class of bioactive constituents from cranberry materials are a type of complex carbohydrate, commonly referred to as soluble dietary fiber. The possible biological significance of these compounds in cranberry received renewed attention in 2010 after first reports that an octasaccharide arabinoxyloglucan, a type of soluble, neutral, hemicellulose oligosaccharide, had been isolated from the urine of swine fed cranberry material [24]. The single, fully characterized arabinoxyloglucan octasaccharide (**1**, Figure 1) identified in this study allowed for the identification of a complex profile of related oligosaccharide constituents in urine fractions with bacterial anti-adhesion properties (Figure 2) [24,25,26]. Trial and error led to the discovery that selectively enriched fractions containing primarily this series of oligosaccharide compounds could be obtained using Sephadex LH-20 column chromatography with 70% ethanol, and that reversed-phase and normal-phase HPLC with photodiode array (PDAD) and evaporative light scattering detection (ELSD) could be used to detect and separate them. Using these methods, a similar complex profile of oligosaccharide components was found in fractions of the cranberry hull extract powder (CHEP (descriptive details in Supplementary Materials)) source material used for the study (Figure 2), and these fractions also had bacterial anti-adhesion properties [24,25,26]. These findings indicated that cranberry-derived oligosaccharides may be excreted unchanged into mammalian urine and may contribute to the anti-adhesion properties observed for urine produced after cranberry consumption [4].

**Figure 3:**
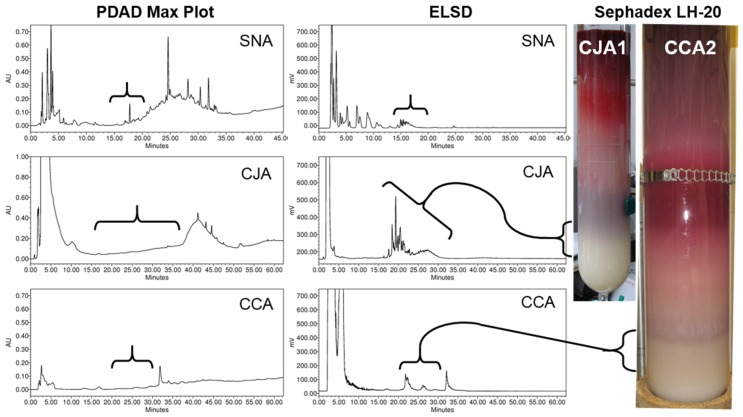
HPLC-PDAD-ELSD chromatographic profiles showing total soluble oligosaccharide content of comparable WBP (SNA), CHEP (CJA), and CJS (CCA) fractions on the Atlantis dC_18_ sorbent, with images of the Sephadex LH-20 separations for CJA1 and CCA2 materials [24,25,26,27,28,29]. Brackets indicate oligosaccharide elution regions and lines connect corresponding materials on the two sorbents. Oligosaccharide components cannot be detected by PDAD but can be detected by ELSD.

Later studies of CHEP and cranberry fruit powder (WBP) that applied the same isolation methods as used in the porcine urine studies led to the isolation and full structural elucidation of two arabinoxyloglucans, hepta- (**2**) and octasaccharides (**3**) (Figure 1) [27,28], with structures related to that of the urinary octasaccharide **1** [25,26]. The ^1^H-NMR spectrum for the purified cranberry octasaccharide **3** was also strikingly similar to that of an additional oligosaccharide component isolated from porcine urine (HF2-2P1t22A17) that could not be fully elucidated due to limited material quantities [26,28] (Figure 2). These observations provided further support for the probable significance of cranberry oligosaccharides for UT health. Subsequent studies, using similar separation and detection methods, identified complex oligosaccharide profiles in both cranberry juice concentrate syrup (CJS) and commercial cranberry juice products that were similar to the oligosaccharide series identified in previously investigated cranberry materials and porcine urine [25,29,30] (Figure 3). 

**Figure 4:**
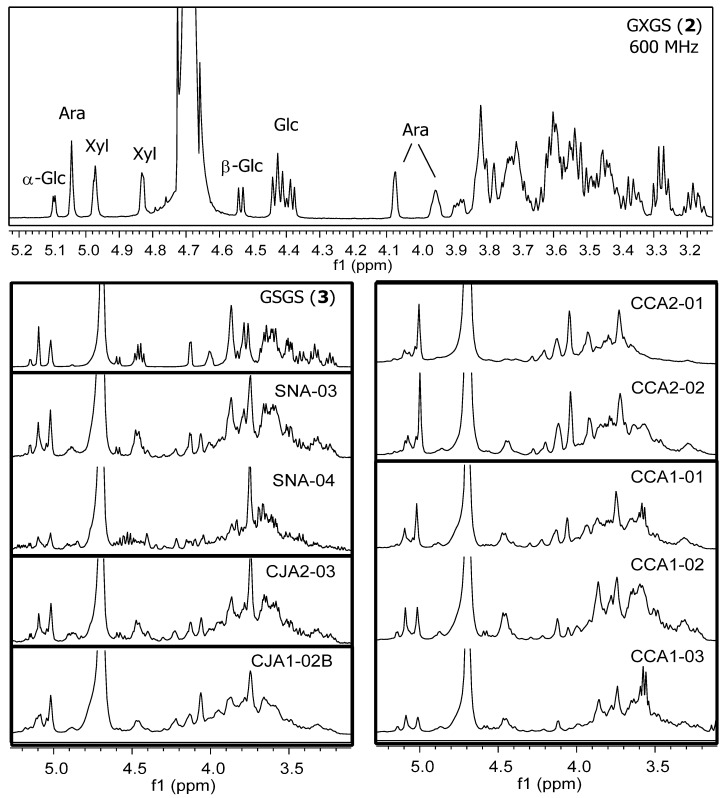
^1^H-NMR spectra (D_2_O, 400 MHz) for oligosaccharide fractions of WBP (SNA-03, SNA-04) [27,28], CHEP (CJA1-02B, CJA2-03) [25,26,28], and CJS (CCA1-01, CCA1-02, CCA1-03, CCA2-01, CCA2-02) [25,29] with anomeric (4.3–5.2 ppm) and other characteristic proton resonances indicated on the spectrum of the GXGS heptasaccharide **2** at 600 MHz [28]. The cranberry GSGS octasaccharide **3** [28] is included to show the similarity of the ^1^H-NMR spectrum at 400 MHz for this purified material to those of complex mixtures containing structurally related compounds. Boxes indicate materials that are fractions from the same chromatographic separations.

Total oligosaccharide fractions from all three materials also yielded similar ^1^H-NMR profiles [24,25,26,27,28,29] although, as observed for the CCA1 (CJS) oligosaccharide fraction series [29], distinct features of these ^1^H-NMR profiles show subtle differences as the total oligosaccharide mixture is further resolved into subfractions (Figure 4).

Several research groups and a growing number of studies have since investigated the structural features and biological properties of cranberry xyloglucans and associated oligosaccharides. The combined findings of these studies suggest the need for a dramatic shift in perspective with regards to the possible bioactive constituents of cranberry materials.

This review will discuss what is currently known regarding the chemistry, occurrence, and biological properties of cranberry complex carbohydrates, especially oligosaccharides, in various cranberry products. As limited information is available regarding the direct effects of cranberry complex carbohydrates on the human microbiota, the bioactivity of these compounds will be discussed partly in the context of the biological properties of related carbohydrates from other dietary sources. Special consideration will be given to the possible significance of cranberry oligosaccharides in regards to the recently recognized UT microbiota in the context of UTI prevention. The overall objective of this review is to provide researchers with an understanding of the new hypotheses that exist pertaining to the biological properties of cranberry complex carbohydrates and the possible significance of these compounds for the health benefits attributed to various cranberry materials. Our hope is that consideration of these new hypotheses may help to resolve at least some of the controversy surrounding the ability of cranberry phytoconstituents to support UT health and prevent UTIs.

### 1.1. Diversity of Cranberry Products

**Figure 5:**
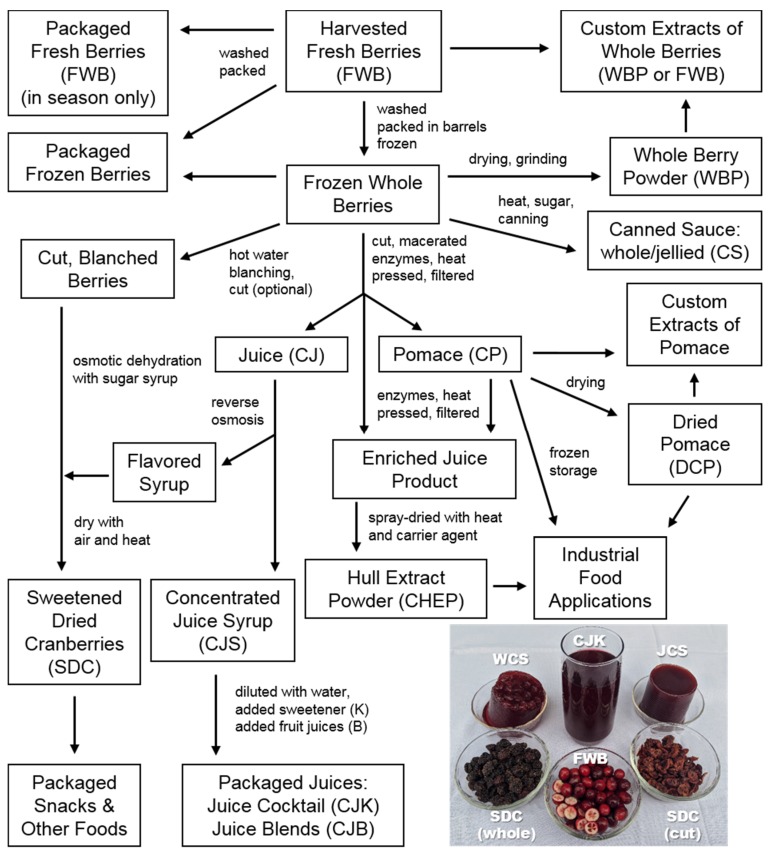
Flow diagram showing generalized processing steps and relative relationships for different cranberry materials. Picture inset shows the appearance of several products that are generally available to consumers and the internal, white flesh (the mesocarp) of fresh whole cranberries. The anthocyanin pigments responsible for the characteristic burgundy color of cranberry products are located in the skin (the epicarp) of fresh cranberries and are dispersed during processing. Figure contents are partly based on personal observations made by the authors during a tour of an Ocean Spray Cranberries, Inc. processing facility in May, 2013.

Multiple types of cranberry products have been used in both clinical investigations and in vitro studies and yet comparative effects of the relative chemical composition on reported bioactivity for different products have rarely been directly considered. The cranberry is a low growing, evergreen shrub that is native to North America. The berries, known as cranberries, are a major agricultural product in areas where it can be cultivated in both the United States and Canada [31,32,33]. When in season, October to December, the fresh berries are widely sold in U.S. grocery stores, but this accounts for a relatively small percentage (3–5%) of overall production [34]. Of the many different types of berries that are considered functional foods, cranberry is somewhat unique in that it is rarely consumed fresh due to the sour, astringent flavor of the berry [2,3]. This has resulted in a wide variety of cranberry products that are available to both researchers and consumers (Table 1, Figure 5), with a range of chemical profiles due to the processing methods used.

The majority of harvested berries are used to produce concentrated juice syrup (CJS) that is then used to produce sweetened (CJK) or blended (CJB) fruit juices [31,35,36,37]. The byproduct of juice production, the residual fruit hulls in the form of pomace, may be used as an animal feed additive or in the production of other foods and nutraceuticals [38,39,40,41]. Whole berries may also be processed into specialty foods or extracts by various methods, and products include sweetened dried cranberries [34,42], canned cranberry sauce [43,44,45], and various foods to which cranberry materials have been added.

Cranberry products are also significant in the dietary supplement market [46]. These products may be derived from any stage of cranberry processing [47] and are most often found as capsules labeled as containing some kind of cranberry material. Dietary supplements are typically powdered or syrup forms of concentrates, extracts, or whole fruit or juice materials. Some of these products may be labeled as standardized to flavonoid content, but labeling may not accurately reflect product composition [48,49,50]. The methods used to derive concentrates or extracts are typically not described or available to consumers, and the drying methods used to obtain the powdered form of a given material are also typically not disclosed. This lack of methodological information makes it impossible to predict what types of compounds may be present in a given product based on labeling. Metabolomics and other chemical profiling studies of cranberry dietary supplements have indicated that many such products may contain adulterants, may have low total cranberry content, or may lack most of the diverse cranberry phytochemicals that are believed to contribute to biological activity [48,49,50,51,52,53].

### 1.2. Processing Methods Influence Product Composition

The multiple forms of processed cranberry materials (Table 1, Figure 5) correspond to multiple variations in resulting physical and chemical composition, as every type of processing has unique effects on the composition of the final product [50,52,54,55,56,57]. Each resulting formulation would therefore provide a different subset of cranberry chemical components and their derivatives within a unique food matrix that may affect biological activity in vivo and in vitro. Use of the suggested acronyms for these various materials as shown in Table 1 and Figure 5 may aid researchers in comparing materials used in future studies.

Different types of cranberry processing physically and chemically separate components of fresh whole berries (FWB) based on solubility and other component characteristics. Juice extraction involves the separation of solid and liquid fruit components by pressing or decanting, followed by centrifugation and filtering, and the liquid may be further filtered in stages to remove suspended solid particulates [37,58,59]. Juice materials would contain primarily water-soluble components such as various flavonoids (pigments and flavor components), mono- and di-saccharides (glucose, fructose, sucrose), organic acids (citric, tartaric, fumaric, and shikimic acid) and a wide range of other small molecules that contribute to flavor [5,35,60,61]. The solid pomace material would consist of cranberry hulls and seeds and contains the berry skins, waxes, insoluble structural polymers and proteins, as well as any compounds adhered to these various physical residues [38,39,62]. Low calorie juice production involves further processing of initial extracted juice materials by proprietary methods, selecting for flavor and pigment components and excluding sugars such as mono- and di-saccharides [63,64]. Specialty processes for nutraceutical preparations, enriched juices, and custom food products lead to further possible variations in final product composition [42,45,47,58,61]. Many of these processing steps may result in soluble oligosaccharides as components of multiple cranberry products and juice materials, and this class of compounds has been identified in juices produced from other fruits such as pears [65]. Intentional enrichment for xyloglucan components or oligosaccharide mixtures may also be used for preparation of nutraceutical products [66].

Processing steps may also change the chemical composition and chemical characteristics of cranberry products in ways similar to those observed for other fruits [49,55,65,67]. Whole fresh berries are typically washed shortly after harvest and then stored frozen for several months prior to processing, during which time cellular structural changes may occur [59,67,68]. Juice is typically extracted from frozen berries that have been cut and macerated while sweetened dried cranberries (SDC) are typically prepared from frozen berries that have been cut [42,69]. The freezing, cutting, and maceration processes physically increases the surface area of fruits by breaking down cell wall components such as cellulose, hemicellulose and lignins [42,58,59,61,68,70,71]. Pectinase enzyme mixtures (such as Klerzyme 150 Pectinase) are typically used with water and heat to biochemically degrade cell wall structures for increased juice production or to produce nutraceutical products [47,58,61,66,72]. The liquid derived from juice production is pasteurized using heat and concentrated to remove water prior to long-term storage. Juice concentration is typically performed using reverse osmosis or countercurrent chromatography to yield a product commonly called “cranberry juice concentrate” (CJS) or “cranberry syrup” with a high solute concentration (50 Brix) [37,61]. Nutraceutical products such as cranberry hull extract powder (CHEP) may be obtained from either frozen, cut, and macerated whole cranberries or from the pomace resulting from standard juice extraction processes [47,56], though the use of whole cranberries is more common. Either starting material is treated with additional enzymes and heat beyond those typically used for juice production [47,56]. After pressing and filtering, the resulting enriched juice extract is typically spray-dried, a process that aerosolizes the liquid or slurry and evaporates the moisture in a heated chamber [55,56,73]. Spray drying is a more economical and faster process than freeze-drying (lyophilization) for the formation of a powder from high-solute, hygroscopic liquid materials, but it involves the use of additional heat and a carrier agent [55,56,64,73]. Drum drying may also be used for some cranberry materials with lower hygroscopic sugar contents, and involves the distribution of the fresh juice or fruit slurry liquid over the surface of a heated drum to create a layer of dried material that may then be ground into a powder [56]. Lyophilization, with the addition of anticaking agents such as silicon dioxide during grinding, may be used to generate powder from fresh whole berries (FWB) or pomace (CP). Frozen grinding in liquid nitrogen and spray or drum drying may also be used to produce WBP and DCP materials [47,55,56,74,75]

Chemical and biological reactions that occur at each processing step also influence the final chemical composition of each cranberry product. Treatments with pectinase and other enzymes cleave polymeric pectins, cellulose, and hemicelluloses found in the whole berries into lower molecular weight (MW) compounds. These relatively low MW plant cell wall components may end up in both pomace and juice based on solubility and interactions with other components of both materials, as has been observed for other fruit juices [65,76,77]. Different types of enzyme treatments with different temperature and time conditions may lead to a variety of effects on the concentration and composition of cleaved monomers or oligomers in a final product. The types of enzymes and methods used may also affect the final concentrations of other phytochemicals released as a result of the degradation of specific structural components [47,58]. Various concentration and drying steps (e.g., reverse osmosis, lyophilization, spray drying) may further modify product composition by changing relative solute concentrations, removing volatile flavor and aroma components [78] through the use of heat and vacuum, and by causing oxidation, degradation, or modification of various components through heat or concentration-dependent chemical reactions [37,55,56,79]. The use of Brix measurements assesses total dissolved soluble solids, but the relative concentrations of individual solutes may vary as a result of multiple factors [80], and a given Brix value does not reflect differences in final product composition across batches. Heat treatments may lead to increased monomeric flavonoid concentrations due to the inactivation of intrinsic degradative enzymes and the degradation of heat-susceptible polyphenolic macromolecules [54,58,61,81]. Reverse osmosis used for juice concentration and the osmotic dehydration process used for the production of sweetened dried cranberries (SDC) may both lead to the unintentional loss of flavor components and other osmotically mobile small molecules from the final product [81,82,83,84], or may result in the transfer of compounds from juice to SDC products [42]. The jelly making process involves heat and the addition of sugar that leads to the crosslinking of pectins and hemicelluloses to form a gel [43,44,45]. The unknown methods applied to cranberry materials for the production of dietary supplements may remove or destroy certain subsets of bioactive components while concentrating for others, but the overall resulting effects on product composition cannot be hypothesized without methodological information [47,50,52,55,56,66].

### 1.3. Common Chemical Components of Cranberry and Related Species

While all cranberry products may have health benefits, CHEP and juices made from CJS are two of the most commonly studied products in terms of UT health. The bioactive compounds present in both materials must therefore survive many of the processing steps described above as well as those unique to the production of both CJS and CHEP. Such compounds would also need to be originally present in whole berry materials (such as WBP) rather than be the products of chemical reactions resulting from specific processing steps. The effects of processing on the chemical composition of the final product are well recognized for flavonoids and other polyphenolic compounds [1,2,3] and may also impact other chemical classes [1,18]. Compounds that remain unchanged through a range of processing steps and that consistently end up in multiple product formulations may therefore be of the most significance in terms of their contributions to the similar biological effects observed for diverse cranberry products.

The observation of similar health benefits for multiple *Vaccinium* products also suggests that a core set of similar compounds may be present in all such materials. Multiple species within the *Vaccinium* genus have been reported to have biological properties similar to those of *V. macrocarpon*. Among the many examples that exist, two include the anti-biofilm properties for lingonberry extracts (*V. vitis-idaea*) [85] and the observed urinary anti-adhesion and UTI-prevention properties for both blueberry (*V. corymbosum*) juice and the high MW components of blueberry juice [86,87]. Blueberry materials have also been attributed with many similar protective effects as cranberry materials with regards to metabolic diseases, inflammation, cardiovascular diseases, and cancer [12,13,88,89].

Studies that have sought to identify chemical differences between *Vaccinium* materials have also indirectly identified common chemical profiles. While different *Vaccinium* species and cultivars undoubtedly have distinct subsets of compounds, an alternate subset of compounds may be similar across species and cultivars. For example, approximately ~6500 individual compounds, 55–57% of the total compounds detected by a UPLC-TOF-MS metabolomics study [90], have been reported to be similar across multiple cranberry cultivars. These values are likely to be underestimates, however, as this study was limited by the inherent limitations of all metabolomics methods [91] and therefore focused on a relatively small subset of all possible phytoconstituents. Comparisons of chemical complexity in relation to bioactivity are further influenced by the disregard typically given to primary metabolites and compounds of relatively high MW (>1000 Da), and by the relative inability of any one detection or analysis method to identify or characterize all chemical components present [92]. Combined, these and other considerations typically result in additional underestimates of chemical similarities between materials.

Many of the compounds identified as unique in a comparative chemical study can be categorized as secondary metabolites or “natural products” rather than as primary metabolites or core structural elements [93]. Secondary metabolites typically have specialized functions within a plant and are therefore more likely to vary as a result of specific environmental and evolutionary pressures [93,94]. These types of compounds also typically have a higher biosynthetic cost of production [93,94]. While secondary metabolites are often the focus of investigations seeking bioactive natural products and have yielded numerous drug products [95,96], compounds classified as primary metabolites or structural compounds can also have significant biological properties and health benefits [76,97]. This is especially the case when bioactivity involves beneficial effects on the human microbiota [98] or other non-lethal effects on bacterial phenotypes, such as compounds with anti-adhesion or other anti-virulence properties [99,100,101].

Primary cell wall components such as cellulose, hemicellulose, and pectins are high MW structural polysaccharides that are likely to possess similar structural features across multiple species within a given genus [77,102,103,104,105]. During fruit ripening and softening, before and after harvest, and during storage, these structural polymers are broken down by natural enzyme cleavage to yield lower MW oligomers with various degrees of polymerization (DP) [34,67,68,77]. Structural polymers may also be affected by commercial enzyme preparations and other degradative processes that occur during fruit juice production and processing, leading to the generation of lower MW oligosaccharides (DP 3–10) [65,77,106]. In the case of cranberry materials, the hydrolysis of pectins may yield acidic pectic oligosaccharides composed of β-galacturonic acid monomers, while the hydrolysis of β-glucan-type hemicellulose polymers would yield oligosaccharides such as the xyloglucans and arabinoxlyglucans, depending on side chain substitution patterns. Whether they are formed as a result of natural enzyme cleavage or commercial enzyme mixtures, these compounds are relatively stable to heat, chemical degradation, and most storage conditions [76,87,103,106]. Structural polysaccharides and their related oligosaccharides may therefore compose a substantial percentage of various fruit and berry products [76,97,104,107]. These types of compounds are commonly described as insoluble and soluble dietary fibers, and are well recognized as having potentially significant impacts on human health [97,98,108,109,110,111].

## 2. Oligosaccharides: Structures, Separations, Occurrence

While the high MW complex carbohydrate components of cranberries are likely to have significant biological properties similar to those of other dietary fibers [108,109,110,111], limited direct information is currently available on cranberry fibers, specifically, in the context of human health. Soluble oligosaccharides will therefore be the primary group of complex carbohydrates discussed further, with reference given to what information is available for some higher MW constituents such as the pectins. Various methods applied to multiple cranberry products have enriched for particular subsets of cranberry oligosaccharide mixtures, including xyloglucans and arabinoxyloglucans, pectic acid oligomers, and, possibly, arabinans and arabinoxylans.

### 2.1. Cranberry Xyloglucans

Xyloglucan oligosaccharides, specifically arabinoxyloglucan oligosaccharides, have been identified in, isolated from, or characterized from multiple cranberry materials. These include commercially obtained juice products [25,30,112], industrially produced CJS [29], non-dialyzable material (NDM) obtained from CJS [113], CHEP [25,26,27,28,112,114,115], and WBP [27,28,30]. The identification of structurally similar oligosaccharides from multiple cranberry products, including whole berries, indicates that these compounds are inherent to cranberry materials.

#### 2.1.1. Structural Features

**Figure 6:**
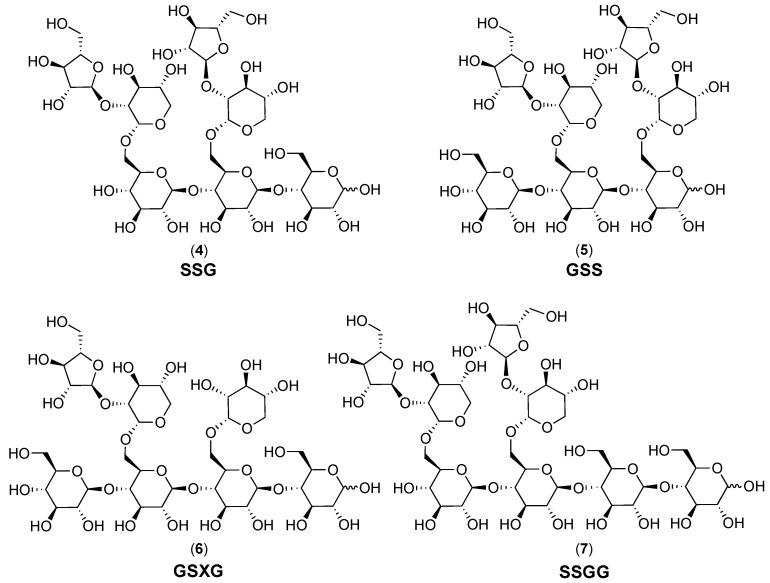
Structures proposed for example hepta- and octasaccharide arabinoxyloglucans identified in CHEP based on MALDI-TOF/TOF MS/MS fragmentation pattern analyses [72,112]: SSG **4** and GSS **5** with *m*/*z* 1055.32 [M + Na]^+^; GSXG **6** with *m*/*z* 1085.33 [M + Na] ^+^; SSGG **7** with *m*/*z* 1217.37 [M + Na]^+^. Absolute configurations are based on monosaccharide identities and the most common anomeric linkages reported for these types of structures and for cranberry oligosaccharides **2** and **3** [28].

The cranberry xyloglucans that have been characterized to date (Figure 1 and Figure 6) contain a backbone of β-(1→4)-linked D-(+)-glucopyranose units that may or may not be substituted at *O*-6 with α-linked d-(+)-xylopyranosyl side chains. Xylosyl side chains may be further substituted at the *O*-2 position with α-linked L-(+)-arabinofuranosyl units [27,28,72,112,114] or arabinosyl units with other linkage positions [114]. An additional side chain constituent may be β-D-(+)-galactose; putative structures linking this monomer to a xylosyl residue have been proposed but have yet to be fully elucidated [27,28,112,114]. Small amounts of β-D-galacturonic acid, α-L-rhamnose, and α-L-fucose have also been detected in oligosaccharide fractions [112], but the connectivity of each monomer has yet to be established. Analysis of MALDI-TOF/TOF MS/MS fragmentation patterns for dominant molecular ions within the total soluble oligosaccharide mixture have led to proposed structures for a series of related compounds; examples of three heptasaccharides **4**–**6** and an octasaccharide **7** from this proposed series are included in Figure 6 [72,112].

The most abundant molecular ion reported for cranberry oligosaccharide mixtures, *m*/*z* 1217 [M + Na]^+^, is believed to indicate a xyloglucan octasaccharide with a Hex_4_Pent_4_ structure [112]. Multiple side chain placements are possible for even this single *m*/*z* feature, although some are believed to be more likely due to preferred enzyme cleavage locations and characteristic fragment ions formed during MALDI-TOF/TOF MS/MS analyses [112,116,117,118]. This octasaccharide is currently believed to be composed of a tetrameric β-(1→4)-linked glucopyranosyl backbone with two α-L-arabinofuranosyl-(1→2)-α-D-xylopyranosyl-(1→6)-linked side chains [112]. These side chains have been reported as being located on either alternate (GSGS structure, **3**, Figure 1, [27,28]) or adjacent (SSGG structure, **7**, Figure 6, [112]) positions on the tetrameric backbone. The octasaccharide isolated from porcine urine after cranberry consumption was found to have yet another type of adjacent side chain placement (GSSG structure, **1**, Figure 1) [25,26].

**Figure 7:**
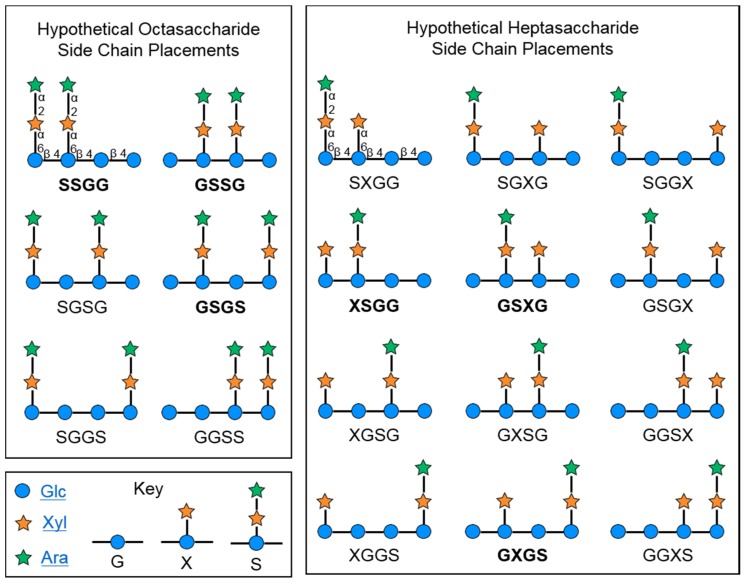
Hypothetical side chain placements for hepta- and octasaccharides (DP 7 and 8) with tetrameric β-glucan backbones and xylosyl and arabinoxylosyl side chains, shown using Symbol Nomenclature for Glycans [119,120] and side chain code abbreviations [116,118]. Reported and proposed structures [26,28,112] are indicated by bolded abbreviations. G: β-D-Glc*p*. X: β-D-Glc*p* with terminal α-D-Xyl*p* at *O*-6. S: β-D-Glc*p* with α-l-Ara*f*-(1→2)-α-D-Xyl*p* at *O*-6.

For even a limited group of hypothetical octasaccharides based on compounds **1** and **3** (Figure 1), with tetrameric β-glucan backbones and a restricted subset of interglycosyl linkages and duplicated side chains, a series of closely related compounds is possible (Figure 7). The set of structural possibilities is expanded when two different side chains are present for a heptasaccharide with a tetrameric β-glucan backbone (Figure 7). This is shown for variations on the theme of compounds **2** (GXGS structure, Figure 1) and **6** (GSXG structure, Figure 6) with α-L-arabinofuranosyl-(1→2)-α-D-xylopyranosyl-(1→6)-linked and α-D-xylopyranosyl-(1→6)-linked side chains. Heptasaccharides and octasaccharides with trimeric (SSG and GSS structures **4** and **5**, Figure 6) or pentameric β-glucan backbones would yield additional possible sequential variations similar to those shown for tetrameric β-glucan backbones. Further complexity and structural variation is introduced by the inclusion of *O*-acetylation [112] at various positions, additional monomers or linkage positions, various combinations of side chains, and combined variations in backbone lengths and side chains to yield oligomers with various degrees of polymerization (DP). Extensive detailed structural work on isolated compounds is therefore needed for all cranberry oligosaccharide components.

#### 2.1.2. Chromatographic Resolution and Aggregation Behavior

Cranberry oligosaccharide mixtures are uniquely difficult to resolve into individual components. Mixtures typically exhibit poor or unexpectedly atypical chromatographic behavior, resulting in broad single elution peaks that appear to be single compounds prior to further investigation. This effect has been observed using multiple sorbents and solvents [25,26,113,114] and several authors have proposed that cranberry xyloglucans form aggregates that contribute to these poor elution profiles [26,29,113,114]. Aggregation behavior may cause mixtures of cranberry xyloglucans to behave as though they are individual, higher MW polymers rather than a series of distinct, structurally related oligomers, resulting in the failure of standard molecular sizing methods to effectively assess the individual MWs of these compounds. The use of high performance size-exclusion chromatography has yet to yield satisfactory results for cranberry oligosaccharide components [72,112,113,114], and individual xyloglucan oligomers from CJS with masses of 900–1500 Da were retained as a mixture in both water and 50% ethanol by dialysis tubing with a MW cut-off of 12–14 kDa [113]. Anti-agglutinating components of CHEP, which are now recognized to include oligosaccharides, were also observed to be retained in aqueous solutions by dialysis membranes with MW cut-off limits of 3, 10, and 30 kDa, while filtrates all lacked anti-agglutination properties [25].

The various solution-state behaviors of xyloglucan oligosaccharides have not been well studied, but hydrophobic and hydrophilic interactions are both believed to affect solution state conformations and inter-and intra-molecular interactions in plant cell walls [103,105,121,122]. Inter- and intra-molecular interactions between water, alcohol, and solute molecules may also lead to the formation of soluble gels and aggregation networks in aqueous solutions with either ethanol or methanol.

Observations from molecular modeling simulations using an example galactoxyloglucan oligosaccharide [123,124] may help explain some of the aggregation behavior observed for cranberry xyloglucans during chromatographic separations with alcohol-water solvent mixtures. The xyloglucan used for modeling studies [123,124] was composed of a 12-unit β-(1→4)-linked glucosyl main chain substituted with α-(1→6)-xylosyl side chains that were either unsubstituted or further substituted with β-(1→2)-galactose. When fully solubilized in water, the model galactoxyloglucan was found to prefer a twisted conformation where the side chains stabilized the backbone through inter-residue hydrogen bonds [123]. This helical backbone conformation, combined with further hydration of the solute by hydrogen-bonded water molecules, effectively limited gelation and promoted solubility by preventing intermolecular hydrophobic and hydrophilic interactions between individual solute molecules.

When 23.6% ethanol was added to the simulation [124], it was found that ethanol molecules aggregated around the xyloglucan structure, effectively displacing water molecules and slowing the shrinking-swelling motion of the xyloglucan that normally occurs in water-only solution [123]. The side chain interactions that stabilized the twisted backbone conformation were apparently unaffected by the addition of ethanol. The decreased flexibility of xyloglucan molecules, however, allowed for additional hydrophobic and hydrophilic associations to form between the backbones and side chains of other individual solute molecules, effectively leading to solute aggregation. The added ethanol molecules were found to interact with solutes mainly through hydrophobic interactions, especially with the hydrophobic plane of xylose residues, and hydrogen bonds with ethanol molecules were formed primarily with the HO-3 of xylose and free water molecules. Water molecules filled the voids within the aggregation network formed by several xyloglucan solute molecules and participated in inter-molecular associations and gel formation.

The formation of soluble gels likely contributed to the elution profile of cranberry oligosaccharides on Sephadex LH-20 in 70% ethanol [28,29], and may have improved the resolution of these components relative to that observed for separations performed with Sephadex LH-20 and only water [112] or lower percentages of ethanol [113]. In contrast, aggregation may also contribute to the poor resolution observed for closely related compounds when C_18_ sorbents are used with 10–25% methanol [26,28,29,112]. Further studies on individual cranberry oligosaccharide molecules and mixed systems will be necessary to fully understand this aggregation phenomenon and its influence on future separation approaches.

Aggregation and non-covalent interactions may contribute to the biological effects of xyloglucan fractions in solution by influencing the interactions of both individual and mixed oligosaccharides with other compounds and cell surface structures [39,125,126]. In particular, phenolic compounds such as flavonoids and proanthocyanidins (PACs) are well known to associate with complex carbohydrates in solution [127,128,129,130]. Solution-state interactions and aggregation effects must therefore be considered for bioactivity studies using mixtures containing oligosaccharides.

### 2.2. Characteristics of Xyloglucan Fractions Used by Various Studies

**Figure 8:**
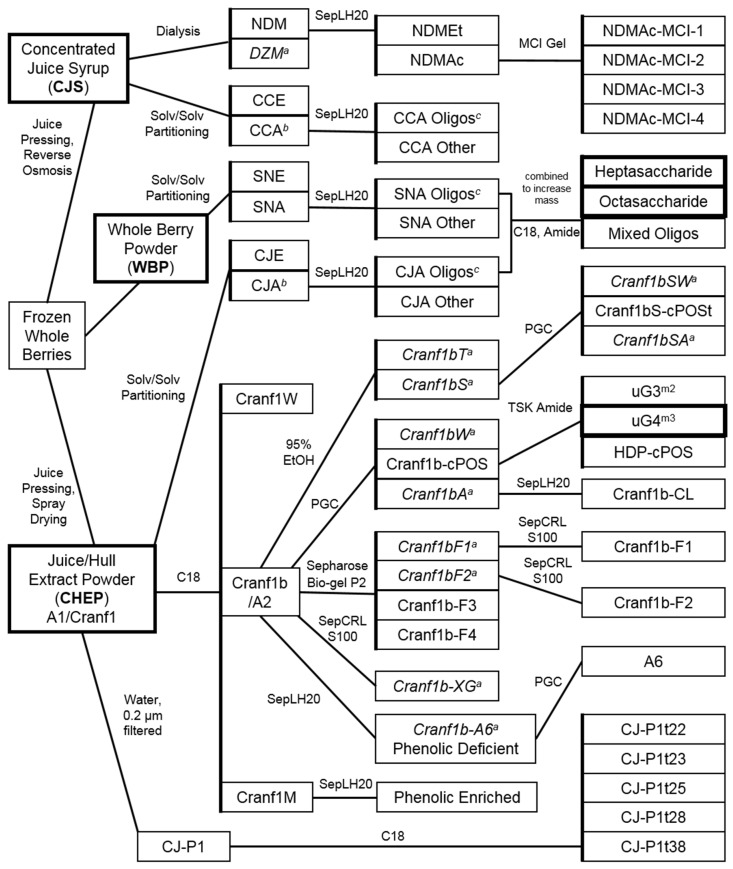
Relationships between cranberry oligosaccharide fractions and parent materials characterized to date by multiple research groups [25,26,27,28,29,72,112,113,114,115,131,132,133]. Summarized separation methods are discussed in the text with additional details included in the Supplementary Materials Table. Original, author-assigned fraction names have been used when possible but some (italics) have been elaborated upon or added to by the current authors to facilitate discussion. Three compounds, a heptasaccharide, an octasaccharide, and a pectic oligosaccharide (uG4^m3^), shown in bold boxes, have been purified and fully elucidated. Thick vertical lines represent fractions from the same separation. ^a^assigned to facilitate discussion. ^b^multiple separations were performed with this material using similar methods. ^c^multiple oligosaccharide subfractions were collected as shown in Figure 9. Abbreviations are as follows: SepLH20 = Sephadex LH-20, PGC = Porous graphitized carbon, SepCRL S100 = Sephacryl S100, EtOH = Ethanol, C18 = C_18_ type sorbent of any manufacture, Amide = polyamide type sorbent, Solv/solv partitioning = ethyl acetate and water in various ratios.

**Figure 9:**
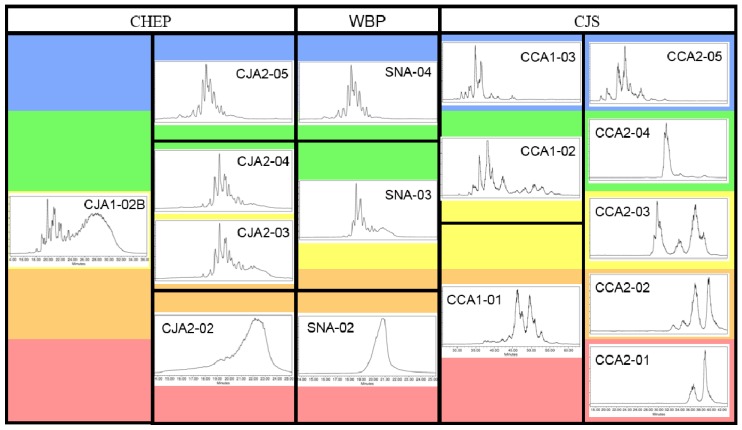
HPLC-ELSD elution profile comparison for Sephadex LH-20 oligosaccharide sub-fractions of WBP [28], CHEP [25,28], and CJS [25,29] materials separated on the same Atlantis dC_18_ column with water to methanol gradients. Various gradient parameters and two different HPLC-ELSD instruments were used to obtain these spectra, resulting in differences in absolute retention times and component resolution between sample sets. Within each respective sample set (e.g., all CCA2 samples), however, separation methods and instruments used are the same. Color bars and boxes indicate approximate fraction composition relative to the CCA2 subfractions as these possessed the highest degree of resolution obtained to date for separations of these mixtures.

The use of multiple fraction names to describe essentially the same material has already led to some confusion regarding which cranberry materials and subfractions can be appropriately compared for even the relatively few studies investigating cranberry oligosaccharides to date [25,26,27,28,29,72,112,113,114,115,131,132,133]. Insufficient material descriptions are also a problem as a minimal standardized method for characterizing and describing oligosaccharide fractions has yet to be established (see Section 2.4.3). The methods used by each set of authors and the structural features described for the fractions obtained to date are summarized below and presented in Figure 8 and Figure 9 with additional method details and fraction descriptions in the Supplementary Materials Table.

#### 2.2.1. Initial Oligosaccharide Separations

At the time the initial cranberry oligosaccharide separation methods were developed, the structures of both urinary and cranberry oligosaccharides were unknown [24,25]. Initial ^1^H-NMR spectroscopic analyses indicated the presence of complex carbohydrates, and analytical and preparative HPLC-PDAD-ELSD separations using multiple sorbents indicated the presence of complex mixtures. ELSD continued to be used by our group as it allowed for semi-quantitative assessments of UV-transparent mixtures with unknown structural features [134,135], and previous attempts to use LCMS had been unsuccessful at detecting this series of uncharacterized (at the time) compounds in samples known to contain them [24]. The application of ELSD to guide preparative HPLC separations was relatively straightforward, and our methods could be directly adapted from analytical to preparative-scale separations by the use of a simple flow-splitter and basic scaling calculations [25,26]. Similar methods were therefore applied to multiple cranberry materials to facilitate comparisons of oligosaccharide composition across samples and individual studies (Table 2). A number of additional separation and detection methods have since been used by other researchers to selectively enrich for oligosaccharide fractions from both CHEP and additional cranberry materials (see Section 2.2.2).

The first cranberry xyloglucan oligosaccharide fractions (CJA1-02, CJA1-02B, and CJA1-03B) were enriched and purified by Coleman et al. [24,25,26] using the same methods that led to the isolation of the GSSG octasaccharide **1** (Figure 1) from porcine urine. This involved dissolving CHEP (referred to as CJ) in water, extracting it with ethyl acetate (CJE: 1% *w*/*w* of CHEP), and then separating the residual aqueous material (CJA: 99% *w*/*w* of CHEP) into oligosaccharide-containing fractions (CJA1 Oligos: 25% *w*/*w* of CHEP) using Sephadex LH-20 with 70% ethanol [24,25,26]. A comparison of these initial Sephadex LH-20 fractions with those later obtained at increased resolution for WBP and CJS materials is shown in Figure 9. An initial direct preparative HPLC-PDAD-ELSD separation of CHEP using the same C_18_ sorbent (Waters Atlantis dC_18_) and solvents as applied to urine fractions also yielded enriched cranberry oligosaccharide fractions (CJ-P1t fractions: 10% *w*/*w* of CHEP) [25,26]. This direct preparative HPLC separation of CHEP yielded only 40% total recovery of the initial material due to sample loss from filtering and split-ELS detection, but verified that these compounds were not affected by previous sample handling, separation methods, or sorbents, and that they exhibited a similar HPLC-PDAD-ELSD elution profile as urinary oligosaccharides. The amounts of oligosaccharide fractions (CJA1 and CJ-P1t materials) collected from both Sephadex LH-20 and preparative C_18_ HPLC separations were sufficient for ^1^H-NMR data acquisition and initial structural comparisons with porcine urinary oligosaccharide fractions, but were inadequate for further purification due to the complexity of the oligosaccharide mixtures present [24,25,26].

Separations of WBP (labeled as SN) and additional CHEP (labeled as CJ) materials were pursued by Auker et al. [27,28], with the objective of obtaining sufficient quantities to allow for the purification and full structural elucidation of component oligosaccharides. Both materials were dissolved in water, centrifuged to remove solids, and then partitioned to yield ethyl acetate (SNE: 0.8% *w*/*w* of WBP; CJE2: 1% *w*/*w* of CHEP) and aqueous soluble fractions (CJA2: 96% *w*/*w* of CHEP). The aqueous portion of the WBP material required an additional solvent-based partitioning with 67% ethanol to precipitate and remove pectins and other insoluble components, yielding fraction SNA (35% *w*/*w* of WBP). Both materials produced early-eluting oligosaccharide-containing fractions on Sephadex LH-20 in 70% ethanol with HPLC-PDAD-ELSD (Figure 2, Figure 3, and Figure 9) and ^1^H-NMR (Figure 4) profiles similar to those of the CJA1 and porcine urinary oligosaccharide fractions [26,28]. The overall yield for the SNA oligosaccharide fractions (SNA Oligos; 18.4% *w*/*w* of WBP) (SNA-02, SNA-03, SNA-04) indicated a lower relative proportion of soluble oligosaccharide components as compared to CHEP, likely due to the higher relative proportion of material that was insoluble in water or 70% ethanol. The overall yield for the CJA2 oligosaccharide fractions (CJA2 Oligos; 38% *w*/*w* of CHEP) (CJA2-02, CJA2-03, CJA2-04, and CJA2-05) indicated improved resolution and additional recovery of the same components collected in the CJA1 separation [25,28].

Selected fractions (CJA2-03 and SNA-03) were further separated in parallel by preparative-scale HPLC-PDAD-ELSD, again using methods previously applied to porcine urine samples [24,25,26]. Fractions collected from the C_18_ sorbent (Waters Atlantis dC_18_) for both CJA2-03 and SNA-03 materials were further separated on a polyamine sorbent (YMC Polyamine II) with 63% acetonitrile to ultimately yield pure compounds from both CJA and SNA materials. Initial ^1^H-NMR data for these purified compounds indicated that both materials contained compounds with the same structures, and appropriate samples were combined to yield sufficient material for further high resolution 1D and 2D NMR spectroscopy and multiple chemical derivatization analyses [28]. Two cranberry oligosaccharides were therefore isolated and fully elucidated from both WBP and CHEP as a GXGS heptasaccharide (**2**) and a GSGS octasaccharide (**3**) (Figure 1). Significant sample loss due to the necessity of using split-ELS detection-collection at each HPLC separation stage yielded overall low sample recoveries; the relative content of these individual oligosaccharides was therefore likely higher than the 0.2–0.5% *w*/*w* recovery of each from both WBP and CHEP materials. The purified and fully elucidated structures for cranberry oligosaccharides were used to further interpret the results of the initial porcine urine studies and allowed for additional tentative assignments to be made for oligosaccharides isolated from porcine urine (Figure 2) [26,28].

Separations of CJS material [25,29] were pursued using similar methods as in previous porcine urine and cranberry studies [24,25,26,27,28] to facilitate comparisons between materials. An initial separation using similar Sephadex LH-20 column parameters as used previously yielded oligosaccharide containing fractions (CCA1-01, CCA1-02, and CCA1-03) with similar component resolution as observed for CJA and SNA separations (Figure 9) [25,26,28,29]. The use of a different large-scale Sephadex LH-20 column with 70% ethanol and altered separation parameters (dimensions, flow rate, loaded sample concentration, fraction recombinations, etc.) led to the collection of oligosaccharide fractions (CCA2-01, CCA2-02, CCA2-03, CCA2-04, and CCA2-05) with improved component resolution relative to those obtained from previous separations (Figure 9) [29]. Formulation differences between source materials (e.g., powder vs. syrup, anti-caking additives, insoluble solid components, etc.) made direct comparisons between parent materials ineffective; the aqueous fractions of all three materials that were soluble in 70% ethanol were therefore compared. The CJS material yielded a lower total percentage of oligosaccharide fractions (CCA Oligos: 4–4.5% *w*/*w* of CCA, Figure 8) relative to the amounts obtained for CJA (CJA Oligos: 26–38% *w*/*w* of CJA) and SNA (SNA Oligos: 53% *w*/*w* of SNA) fractions. The cause for these distinct overall percentages of soluble oligosaccharides in different source materials has yet to be established but it may be due to both product formulation and processing (see Section 1.2). Improved removal of non-oligosaccharide components from the CCA fractions may also have reduced apparent yields.

Combined, our assessments of oligosaccharide-containing subfractions from CHEP, WBP, and CJS materials indicate that processing effects may significantly influence total oligosaccharide content in the resulting cranberry materials. The presence of similar arabinoxyloglucan oligosaccharides in all three materials, however, as observed by HPLC-ELSD (Figure 3 and Figure 9) and ^1^H-NMR data (Figure 4), supports the hypothesis that these compounds are a product of hemicellulose breakdown during fruit ripening [103,136,137], and the relative complexity of the oligosaccharide elution profiles observed for each material may correspond to the relative complexity of the component oligosaccharides. For example, WBP-derived materials may contain soluble oligosaccharides and other complex carbohydrate components that end up in pomace material rather than in CHEP or CJS materials (Figure 5). Oligosaccharides present in fresh pressed CJ material may be removed from CJS during the osmotic concentration process and end up in SDC materials (Figure 5). Enzymatic treatments during processing may also influence relative oligosaccharide concentrations for both CHEP and CJS. Extensive quantitative studies using purified cranberry oligosaccharide standards would therefore be required to effectively assess the distributions of cranberry oligosaccharides across these and other cranberry products.

#### 2.2.2. Targeted Oligosaccharide Separations

Prior knowledge regarding the general nature and presence of oligosaccharides in cranberry materials [24,72,131] has enabled multiple research groups to pursue separations and analyses using methods specifically targeted to the enrichment or detection of oligosaccharides and other complex carbohydrates. The use of MALDI-TOF/TOF MS/MS techniques and extensive fragmentation pattern analyses has led to the proposal of possible structures for a number of major components within the cranberry xyloglucan series by multiple authors, but additional purified xyloglucans have yet to be obtained in sufficient quantity for full structural analysis and individual bioactivity studies. Most studies, to date, have focused on the characterization and testing of oligosaccharide mixtures due, in part, to the difficulty of resolving individual components (see Section 2.1.2). In general, oligosaccharide chromatographic profiles and ^1^H-NMR data reported for the studies discussed below are similar to those discussed in Section 2.2.1. The methods used in the studies described below are summarized in Table 3 with additional details in the Supplementary Materials Table.

Hotchkiss et al. [72,112] described the composition of two xyloglucan containing fractions, A2 and A6, obtained from CHEP material (named A1 and described as “hull enzyme-treated concentrate fraction”) [112]. The CHEP/A1 material was separated via flash cartridge chromatography using a C_18_ substrate eluted sequentially with water, 15% methanol, and 100% methanol to yield A2 (24% *w*/*w* of CHEP) as the 15% methanol fraction. Yields were not given for the other fractions obtained from the separation. The A2 fraction was partially characterized as containing arabinoxyloglucans with small amounts of phenolic pigments, and was further separated using Sephadex LH-20 with water to remove these phenolic compounds and yield the A6 material. The A6 fraction was initially described as containing "neutral, sugar-rich, low-viscosity, low-MW polysaccharides" [112]. Further characterization, however, indicated it contained compounds with DP 5–13 corresponding to MWs [M + Na]^+^ of individual oligosaccharides ranging from *m*/*z* 790–1700 with some larger constituents up to *m*/*z* 2400 [112]. Fragmentation patterns and NMR data also indicated the presence of methyl and acetyl groups on various xyloglucan and arabinoxyloglucan oligosaccharides but confirmed structures containing these substituents have yet to be fully elucidated. The putative structures proposed included primarily oligomers with backbones of 2–5 hexosyl units with side chains composed of 1–9 combined pentosyl and hexosyl units [112]. High performance anion-exchange chromatography with pulsed amperometric detection of both fractions A2 and A6 showed the presence of a complex series of oligosaccharides [112] similar to that observed by our HPLC-ELSD analyses (Figure 3 and Figure 9).

The xyloglucan fractions used by Sun et al. [114] were initially obtained using the same source material, sample preparation, and separation methods as described by Hotchkiss et al. [112]. Initial fractions from C_18_ flash cartridge chromatography of the source material (Cranf1 = A1 = CHEP) were, however, labeled differently, as Cranf1W (100% water: 38% *w*/*w* of CHEP), Cranf1b = A2 (15% methanol: 24% *w*/*w* of CHEP), and Cranf1M (100% methanol: 28% *w*/*w* of CHEP). These authors [114] further separated the Cranf1b/A2 material using anion exchange chromatography (Sepharose Q XL 16/10) eluted with 100% water (Cranf1bF1) and 0.1 M NaCl in water (Cranf1bF2), with subsequent separation of both fractions over Bio-gel P2 and Sephacryl S-100 HR sorbents to remove salts and residual phenolics. Of the two enriched oligosaccharide subfractions (Cranf1b-F1: 14% *w*/*w* of CHEP and Cranf1b-F2: 4% *w*/*w* of CHEP), only Cranf1b-F2 was characterized further by chemical and spectroscopic methods similar to those reported previously (MALDI-TOF-MS, GC-MS, and NMR) [112], with the inclusion of glycosyl linkage analysis. Results indicated a mixture of similar arabinoxyloglucan components as previously detected [112]. Notable differences were that the authors [114] identified additional α-(1→3,5)-linked, α-(1→3)-linked and α-(1→5)-linked arabinofuranosyl residues as well as a higher molar percentage of arabinose (56%) than reported in previous studies [28,112]. This may indicate the presence of free arabinan branched oligomers, or xyloglucans with arabinosyl side chains that are branched or longer than observed previously for other cranberry oligosaccharide mixtures. This observed difference may be due to a higher relative concentration of a particular subset of cranberry oligosaccharides resulting from the fractionation methods used. These authors [114] also generally assigned the observed terminal β-(1→2)-galactosyl moieties to xylosyl side chain residues but individual structures containing this feature have yet to be fully elucidated.

Continued investigations by Sun et al. [115] further separated the cranberry xyloglucan mixture (Cranf1b) using a porous graphitized carbon cartridge with 30% acetonitrile, followed by a C_18_ SPE cartridge eluted with water to remove residual monosaccharides, salts and other minor components and yield fraction Cranf1b-CL (18% *w*/*w* of CHEP). This material was analyzed using ^1^H-NMR spectroscopy and MALDI-TOF MS and by chemical characterization. Cranf1b-CL was reported to contain four major monosaccharides (25% arabinose, 5% galactose, 47% glucose, and 23% xylose) and the MALDI-TOF MS spectrum indicated a series of oligomers with 3–5 hexosyl backbone units and 3–5 side chain pentosyl units with DP 6–9. This material had approximately the same monosaccharide ratio (1:1:2 arabinose: xylose: glucose) as that reported previously for highly enriched arabinoxyloglucan fractions [28,112]. The presence of galactose as a minor constituent was reported, though the authors stated that the fraction was free from monosaccharide components. This observation supports the covalent linkage of galactosyl units to arabinoxyloglucan or xyloglucan oligomers but the authors did not comment further on this structural feature [115].

The cranberry xyloglucan mixture (Cranf1b) used by Ozcan et al. [131] was the same as that used in previous studies [112,114,115]. These authors used size exclusion chromatography (HiPrep Sephacryl S-100 HR 16/60) with aqueous isocratic elution to further enrich for xyloglucan components. The resulting xyloglucan mixture (Cranf1b-XG) was characterized by ^1^H-NMR and MALDI-TOF MS data and reported to lack polyphenol constituents and contain arabinoxyloglucan oligomers of DP 6–9 with backbones of 3–5 hexosyl units and side chains composed of 2–4 pentosyl units [131]. Further structural characterization was not included.

A report by Neto et al. [113] indicated that oligosaccharides are a component of what has previously been described as non-dialyzable material (NDM: 6 g/L of CJS) or as a high MW fraction of cranberry juice concentrate (CJS). Fractionation of NDM (~1.5 g) over Sephadex LH-20 with two solvents yielded two fractions, the first (NDMEt: 74% *w*/*w* of NDM; ~4.6 g/L of CJS) contained primarily oligosaccharides and eluted with 50% ethanol, while the second (NDMAc: 22% *w*/*w* of NDM; ~1.3 g/L of CJS) contained primarily oligomeric polyphenols (such as A-type PACs) and eluted with 75% acetone. Subsequent analyses indicated that both fractions contained minor amounts of other compounds in addition to oligosaccharides and polyphenols. Total phenolic analysis by the Folin-Ciocalteu method with procyanidins A2 and B2 as standards indicated that the NDMEt material contained 9–19% phenolic content, suggesting the presence of low amounts of polyphenols or other reducing substances. In contrast, the NDMAc material contained 52–74% phenolic content, indicating that the majority of the total phenolic content of NDM was present in this fraction. The additional presence of small amounts of oligosaccharides and other compounds in NDMAc was confirmed by subsequent separations and analyses (NDMAc-MCI fractions; Figure 8). The NDMet fraction was tested in biofilm formation and bacterial co-aggregation assays (see Section 4.2) and was characterized by MALDI-TOF MS but was not further separated. The MALDI-TOF MS spectrum of the NDMEt material contained what the authors described as a “regular pattern of masses consistent with increasing numbers of hexose and pentose units.” This series of oligomers ranged in MW from ~900–1500 Da with DP 6–10, similar to a portion of the CHEP oligosaccharide material reported by Hotchkiss et al. [112]. A portion of the mass fragments identified in the NDMEt material was also identified in the NDMAc-MCI-3 fraction along with mass fragments characteristic of polyphenols. The authors [113] did not perform monosaccharide analysis on the NDMEt material, but individual masses were associated with 4–6 hexosyl units and 2–4 pentosyl units, consistent with the β-glucan backbones and xylosyl or arabinoxylosyl side chains reported previously [28,112].

O’Connor et al. [132] used cranberry materials generated from a mixture of freshly harvested cranberry cultivars. They stated that “whole cranberry powder was prepared by grinding cranberries frozen in liquid nitrogen into a powder” but did not provide relative mass quantities or recoveries for any of their described separation steps. From this WBP material they generated “phenolic-enriched cranberry extract,” and “phenolic-deficient cranberry extract” using methods that appeared similar to those reported previously [112], although relevant references were not cited. The material the authors [132] describe as “phenolic-deficient” can also be described as oligosaccharide-enriched, though the authors did not use this phrasing. They also reported the inclusion of seven different oligosaccharide fractions for testing in an antimicrobial assay but few details were provided regarding the relative composition of these fractions or the specific methods by which they were prepared [132]. The materials were described as “crude” and “refined” and as “oligosaccharide” but no indication was given as to relative purity or monomeric composition [132]. The crude and refined fractions were reported to have been obtained from “cranberry juice powder” and “cranberry concentrate” while the “oligosaccharide” materials were described as being derived from “cranberry extract,” “cranberry concentrate,” and “cranberry pomace/ground pomace.” All of these material descriptions are ambiguous with regards to the applied processing steps (see Section 1.2 and Section 2.4.2). As the main objective of the study was to identify cranberry compounds with growth inhibitory properties, further investigation of the cranberry oligosaccharide components was not pursued and insufficient information was provided to effectively compare the included oligosaccharide-containing cranberry materials to those of other studies.

### 2.3. Cranberry Pectins: Occurrence and Structures

Cranberry pectins have yet to be thoroughly investigated for structural features or biological properties. Some general structural features and properties of cranberry pectins have, however, been reported [43,44,45,72,112,139] and one study has reported structures for pectic oligosaccharides associated with the cranberry oligosaccharide material (Cranf1b) investigated in previous studies [133].

Sufficient amounts of pectin are present in whole fresh cranberries to enable the formation of a gel with the addition of only sugar and heat, though the acidity of cranberries is known to have an effect on gel formation [43,44,45,139]. Commercial cranberry juice production is described as requiring sequential hot (50 °C for 1 h) pectinase macerations prior to pressing and filtering in order to obtain the best juice yield [58,61,72], suggesting that pectin is a significant component of the berry structure that must be overcome during juice production.

As enzyme and heat treatments are known to affect pectin structures [77], further studies are needed to fully characterize the content and structural properties of pectins present in various cranberry materials. The use of commercial pectinase enzymes to degrade pectins during cranberry juice processing may result in increased levels of galacturonic acid in juices and juice products. Pectic oligosaccharides resulting from pectinase treatments may end up in either CJS, CHEP or pomace (CP, DCP) materials depending on their solubility and molecular size and the enzyme treatments employed. Cranberry pomace has been reported to contain low levels of polygalacturonic acid pectic polymers [140] and 10–12% acid-extractable pectin, with relatively low proportions (<1%) of water-soluble, chelator-soluble, and hydroxide-soluble pectin [141,142]. Pectin components with various structures are also likely to be a significant contribute to the fiber content of SDC products. Combined, these observations suggest that multiple types of pectic oligosaccharides may be present in different cranberry materials including those derived from ripe whole berries as a result of intrinsic enzyme activity.

Popov et al. [143] isolated a pectic polysaccharide from fresh fruit of the small cranberry (*Vaccinium oxycoccus*). As this species is closely related to *V. macrocarpon*, core structural elements such as pectins may share similar structural features. The pectic polysaccharide was extracted with aqueous ammonium oxalate and partially characterized as containing 82% galacturonic acid, 8% arabinose, 5% glucose, 3% galactose, and 1.5% rhamnose, with a MW of 100–300 kDa. Detailed isolation and structural elucidation methods were not provided and are assumed to be similar to those used in the reference [144] cited by the authors. Linkage positions and other specific structural features were not reported.

The CHEP material (labeled as A1) has been described as containing homogalacturonan-rich pectic polysaccharides as well as other polysaccharides [112]. Monosaccharide analysis indicated that it contained molar fractions of 49.4% galacturonic acid, 25.4% glucose, 10.7% arabinose, 7.4% galactose, and 6.6% xylose, with small amounts (<0.3% each) of rhamnose, fucose, and glucuronic acid. As the CHEP/A1 material contains the A6 arabinoxyloglucan-containing material, these reported molar percentages likely reflect a complex mixture of both pectic and xyloglucan substances as well as other possible oligosaccharides such as arabinans [114]. Differences in the molar percentages reported for the mixed *V. macrocarpon* CHEP fraction and the *V. oxycoccus* isolated pectin material cannot be quantitatively compared, but the two materials are similar in the dominance of galacturonic acid and the presence of glucose, arabinose, and galactose [112,143]. Future studies may be able to resolve the species-specific structural variability of cranberry pectins.

**Figure 10:**
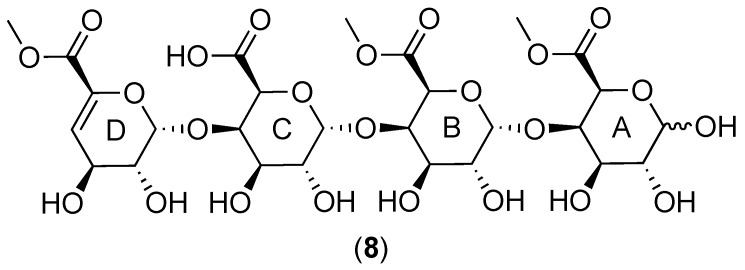
Structure of the pectic tetrasaccharide uG4^m3^ **8**, an unsaturated methyl polygalacturonoate, isolated from CHEP material [133].

Sun et al. [133] observed that the xyloglucan-containing Cranf1b material (from CHEP) was able to induce bacterial colony formation when added to the surface of an agar plate containing quiescent *E. coli* CFT073. Bioassay-guided fractionation led to the identification of a Cranf1b subfraction, Cranf1b-cPOS, that retained this property. Three minor constituents of Cranf1b-cPOS were identified as previously reported iridoid glucosides. These compounds were separated from the Cranf1b-cPOS material and found to be inactive in the quiescence assay while the resulting Cranf1bS-cPOS-t fraction retained the ability to reverse quiescence and persister cell formation. Monosaccharide analysis of Cranf1b-cPOS indicated that it contained only galacturonic acid units, consistent with the dominance of this monomer in other cranberry pectin analyses [112,143]. This material was further fractionated by HPLC to obtain two compounds and the fraction HDP-cPOS, composed of a series of unsaturated methyl polygalacturonoates with DP 3–8 and one or two free carboxylic acid groups. The two isolated compounds, with three (uG3^m2^) and four (uG4^m3^) indices of hydrogen deficiency and two or three methyl esters, respectively, were structurally characterized by HR-ESI-MS/MS and NMR spectroscopy. The structure of uG4^m3^ (**8**, Figure 10) contained the free carboxylic acid group at the C-5 position of ring C and a C-4-C-5 α,β–unsaturated carboxylic acid methyl ester for ring D. Anomeric carbons were depicted with α-(1→4)-linkages, consistent with the predominant structures of homogalacturonan-type pectins, but glycosyl linkage analyses were not included in the study so this absolute configuration has yet to be confirmed. Comparisons of 2D NMR spectra indicated that the structural features of uG4^m3^ were representative for the Cranf1b-cPOS unsaturated methyl poly-galacturonoate series. The authors [133] observed that the formation of the 4,5-unsaturated bond in the Cranf1b-cPOS oligomers may have been due to eliminative cleavage resulting from the specific types of enzymes used to commercially process cranberry materials, further supporting the need for additional research into cranberry pectin structures and the effects of processing (see Section 1.2).

### 2.4. The Hidden Occurrence of Oligosaccharides in Cranberry Materials

Oligosaccharides are likely to be present to different degrees in all types of cranberry materials. Higher MW carbohydrate polymers are additionally likely to be present in whole berry materials, whole berry powder (WBP), sweetened-dried cranberries (SDC), and the pomace (CP) resulting from juice production. While cranberry oligosaccharides have yet to be directly quantified in various materials due to a lack of available standards, initial estimates of total oligosaccharide content can be made based on the yield results of several studies (see Section 2.2). As neutral oligosaccharides are difficult to detect if suitable methods are not applied, the unrecognized presence of these compounds may influence observed results for both in vitro and in vivo studies. The confirmed presence or absence of oligosaccharides in various cranberry materials needs to be reported in future studies and some suggested minimal reporting requirements are included in Section 2.4.3.

#### 2.4.1. Oligosaccharide Content Estimates

An appropriate nutritional category does not yet facilitate the effective quantification and reporting of soluble oligosaccharides in various food products [145]. Composition descriptions and product labeling may indicate values for total content of carbohydrates, sugars, or fibers, but these values are not further subdivided in a way that would enable the assessment of oligosaccharide content. Compositional descriptions for commercially labeled juice products (e.g., “18 g total carbohydrate of which 9 g were sugars” [146]) are equally unhelpful as the “sugar” portion of a label may contain both intrinsic and added “sugars” of various types, including glucose, sucrose, and high fructose corn syrup, among others.

Lyophilized fresh cranberries have been reported to contain, on a dry matter basis, 94.8% carbohydrates (31.4% sugar + 63.4% polysaccharides), 35.7% dietary fiber (some of which is carbohydrate), 3% protein, 1% fat, and 1.2% minerals [126] but these values total to 136%. Materials such as “NutriCran90” used in several studies [147] have been described as containing “at least 88% carbohydrates, 30% organic acids, and 2.0–3.8% total phenolic compounds,” giving a total percentage of 122%. These overabundant total percentages may be due to the duplicated detection of compounds such as oligosaccharides variously as carbohydrates, sugars, polysaccharides or fibers, due to different characteristic structural features and the content assessment methods used. Direct oligosaccharide assessment of lyophilized WBP has yielded a total soluble oligosaccharide content of ~18% *w*/*w* (see Section 2.2.1) [28] and the portion of the WBP that was insoluble in water or 70% ethanol may represent additional, higher DP fiber components such as pectin, hemicellulose, and cellulose.

General chemical analysis of CHEP provides relative percentages for multiple classes of compounds, but the values given account for only 85% total content [148,149], leaving 15% of the material content unknown. Even if 10% of the material mass is a carrier or anticaking agent [148,149], this still leaves 5% of the dry weight unaccounted for. In contrast, direct assessments of oligosaccharide content in CHEP range from 16–38% *w*/*w* oligosaccharides (see Section 2.2). As lower values may represent sample loss during separations or enrichment variations as well as differences in product composition, a conservative approximation for the dry weight content of soluble oligosaccharides in CHEP would therefore be ~20% *w*/*w* (20 g/100 g).

Juice materials prepared by different manufacturers may contain different relative proportions of low DP, soluble oligosaccharides and hydrolyzed pectins depending on manufacturer methods (see Section 1.2) but these differences have yet to be thoroughly characterized. Initial investigations of commercially available juice products [25,30,112,113] indicate these materials contain detectable oligosaccharide components, and that total oligosaccharide content may vary in proportion to relative CJS concentration [25,30,112]. Juice products labeled as containing 100% cranberry juice (from CJS) may, however, contain low overall solute concentrations, and therefore low oligosaccharide concentrations, depending on the amount of water used to dilute CJS [25,30]. The oligosaccharide content of CJS also influences the oligosaccharide content of the final juice product, and this concentration may vary relative to the total solute concentration used to establish the 50 Brix standard measurements [80]. Values reported by Neto et al. [113] for the recovery of oligosaccharides from NDM can be extrapolated to provide an approximate concentration of 4 g total oligosaccharides (with some associated non-oligosaccharide components) in 1 L of CJS. Depending on product formulation and relative solute concentrations, these values may correspond to ~1 g of total oligosaccharide mixture for 1 L of 27% cranberry juice cocktail (CJK) made from CJS. Direct assessments of oligosaccharide content in CJS indicate that these compounds are present at lower concentrations (~4.5% *w*/*w* of CCA) than in comparable fractions from CHEP or WBP (see Section 2.2.1), but the proportion of total soluble oligosaccharide content relative to the total dissolved solids of CJS has yet to be quantitatively assessed across batches or production years. These initial estimates provide an approximate starting point for future studies but direct quantification is needed.

Limited information on the oligosaccharide content of commercially available dietary supplements indicates that product formulation and processing may significantly impact oligosaccharide content (see Section 1.2) [25,30]. No information is yet available regarding the total soluble, neutral oligosaccharide content of FWB, CP or DCP, SDC, or cranberry sauces (WCS or JCS). In an investigation of the effects of SDC consumption on urinary proteome and gut microbiota profiles [150], each 42 g serving of the SDC mixture used was reported to contain 33.12 g total carbohydrates with 2 g fiber and 29.02 g sugars, as established by standard USDA nutritional assessment methods. The relationship between these standard values and oligosaccharide content is unknown, as this class of compounds may be classified as both a fiber and a sugar, but it is notable that a portion (2.1 g) of the total carbohydrate content is unaccounted for. All materials derived from whole cranberries are highly likely to contain soluble oligosaccharides, as well as higher DP oligomers than found in juice-derived materials and structural polymers.

#### 2.4.2. Composition Ambiguity for Cranberry Products

The wide diversity of cranberry products with a range of total oligosaccharide contents is further complicated by the use of multiple names, labels, and ambiguous or confusing descriptions given for the cranberry materials used in many research studies. The most common product used by researchers appears to be CHEP and yet this material is described in many ways and the exact methods and starting materials used to obtain it are not always reported. Ambiguous descriptions that exclude the source, such as “cranberry powder” or “cranberry extract powder,” and “cranberry extract,” may actually refer to “cranberry hull extract powder (CHEP),” but this assumption cannot be definitively confirmed with the limited information provided in most studies. The phrase “cranberry extract” is additionally ambiguous if methods are not reported, as it could describe material obtained from a juice (water) extraction from solid berry material or an organic solvent-based extraction from any type of cranberry material. Descriptions of material as “cranberry juice powder” may refer to either CHEP or dried juice products though the latter scenario is less likely. The drying methods used to obtain an actual juice powder must also be reported as these may affect composition. Descriptions such as “cranberry powder” do not specify whether the powder was obtained from a specialty production process (CHEP), whole berries (WBP), or some other type of extraction and powdering process. Compositional differences between CJS, CHEP and WBP would be significant, with neutral and pectic polysaccharides and higher DP oligosaccharides being present in whole berry materials, and differences for all three materials with regards to relative concentrations of lower MW oligosaccharides resulting from different enzymatic cleavage processes. Descriptions of material as “freeze-dried cranberry powder” are doubly ambiguous as they may refer to lyophilized whole berries that have been ground, or may be inaccurate descriptions of CHEP (this material is typically spray-dried rather than lyophilized). In addition to confusion over product origin and initial processing, distinction between the two drying methods is significant as each would have different effects on product chemical profiles (see Section 1.2).

Unrecognized oligosaccharides may be present in a variety of cranberry fractions generated from this range of ambiguously described starting materials and may therefore influence observed study results. Numerous chromatographic separation methods may inadvertently enrich for oligosaccharides in conjunction with other components without the awareness of the researchers. Many standard chromatographic separations used for both detection and isolation employ UV or LCMS-based detection methods, both of which are largely insensitive to the presence of cranberry xyloglucans and other neutral oligosaccharides (see Section 2 and Section 3.3) [151]. Even fractions and materials described as “not containing carbohydrates” may contain oligosaccharides, as methods for establishing carbohydrate content are often not disclosed by researchers [152,153]. Generally accepted, chemical-based methods used to assess the presence of carbohydrates often detect reducing monosaccharides or disaccharides and may therefore give false negative results for fractions containing higher DP neutral oligosaccharides [25]. Oligosaccharide-appropriate chemical detection methods must therefore be used for accurate results [114,131,138]. The use of appropriate technical terminology is also important, as fractions described as “sugar free” [115] may be lacking in monosaccharides but may still contain significant amounts of oligosaccharides. Partially characterized cranberry materials or fractions that have not been assessed by ^1^H-NMR spectroscopy, and which may contain polar compounds, are therefore highly likely to contain oligosaccharides.

Researchers may include compositional analyses of their chosen study material for a subset of chemical components, typically flavonoids or other phenolics [149,154], but these classes of compounds make up a relatively small overall percentage (<5% *w*/*w*) of most available cranberry materials [1,148]. Cranberries are often described as having a “high total phenolic content” but this type of description is only accurate in relation to other fruits and foods (e.g., cranberry has a higher total phenolic content than strawberry) [60], not in relation to other compounds present in the cranberry itself. Phenolic-enriched materials may contain higher relative proportions of compounds such as flavonoids and PACs than found in original cranberry materials, but may still contain relatively high proportions of complex carbohydrates and other cranberry components, depending on the enrichment methods used [e.g., 113 vs. 151]. Various assessments may characterize the total phenolic content of phenolic-enriched fractions, but such quantifications are based on equivalency estimates and often cannot account for the total mass of all components present in a given fraction [113,149], possibly indicating that the residual portion is non-phenolic. PAC-enriched and PAC-standardized materials are typically described as “spray-dried cranberry concentrate powder standardized to 2.7% PACs,” or as “standardized cranberry extract containing 37 mg PACs” [155,156], and these descriptions are consistent with CHEP as the source material. Conservative estimates of total oligosaccharide content in CHEP (20% *w*/*w*; 20 g/100 g) (see Section 2.4.1) are at least 7x higher than amounts typically reported for the commonly cited total PAC content (2.7%; 2.7 g/100 g) [148,149], and, even with these approximations, 77% of the material composition would still be unaccounted for. Regardless of the starting source or probable oligosaccharide content, it must be acknowledged that materials with PAC concentrations of 2.7% also contain significant proportions (97.3%) of additional cranberry components.

The PAC-enriched cranberry materials used in various clinical studies are also likely to contain significant amounts of oligosaccharides and other cranberry compounds. For example, the 37 mg of PACs reported by Howell et al. [155,156] is presented to study participants in the form of 2 × 500 mg capsules. These values translate to 3.6% PACs and 96.4% of other cranberry compounds or added maltodextrin [155]. The base cranberry materials used to create these enriched PAC-standardized materials include fractions described as “cranberry aqueous extract,” “cranberry hydro-ethanolic extract” or a blend of these two types of extracts [155]. These material descriptions are again consistent with CHEP as the original starting material and oligosaccharides have been found in both water- and ethanol-based extracts of several cranberry source materials (see Section 2.2). No study has yet to directly analyze the total oligosaccharide content for these enriched PAC-standardized materials but, based on conservative estimates for CHEP oligosaccharide content, at least 20% or 200 mg of oligosaccharides may be administered along with the 37 mg of PACs in a 1000 mg dose of the described cranberry materials. Approximately 760 mg of additional cranberry compounds would also be present in such a material and all components present may influence the observed clinical trial results.

#### 2.4.3. Oligosaccharide Content Reporting

The chemical nature of oligosaccharides and their tendency to remain undetected requires that minimum standards be established for both describing and reporting the oligosaccharide content of various cranberry materials. The following are some suggestions based on our observations of the current cranberry oligosaccharide literature. Many researchers investigating cranberry materials may not be familiar with the detailed chemical analysis of complex carbohydrates or may not have access to advanced structural characterization resources such as MALDI-TOF MS or NMR instruments. Fortunately, such familiarity and resources would not be required to confirm the presence or absence of this class of compounds. Those seeking additional knowledge regarding the chromatographic and spectroscopic methods used by complex carbohydrate experts for oligosaccharide structural elucidation are referred to other publications on the topic [116,120,157]. It is our hope that future cranberry researchers will adopt and possibly improve upon the suggested characterization and description standards outlined below:

(A) Minimum requirements (1–3) for oligosaccharide characterization and content descriptions with additional (4) characterization if possible:
  (1) Identification of possible monosaccharide composition using one of the following methods:
    (a) Monosaccharide composition analysis suitable for neutral or pectic polysaccharides and oligosaccharides to give relative molar percentages of monosaccharide constituents.
    (b) ^1^H-NMR spectrum (D_2_O) showing distinct anomeric signals corresponding to oligosaccharides previously characterized from cranberry materials.
  (2) Full description of the source material and separation methods
    (a) Detailed description of the cranberry source material using standardized terminology (Table 1, Figure 5) and additional information as necessary for clarity and reproducibility.
    (b) Inclusion of sufficient separation details (including yields and accurate sorbent descriptions) necessary for another researcher to repeat the methods used.
  (3) Reported presence or absence of additional possible constituents
    (a) A PDAD max plot UV spectrum will show the presence or absence of trace amounts of organic acids and phenolic compounds, such as flavonoids and PACs. A UV spectrum would also indicate the presence of uronic acids such as those present in pectic oligosaccharides.
    (b) Visual description of the dried material. Trace amounts of anthocyanins can be visually detected as a slight pinkish or purplish color when the material is dried.
  (4) Additional characterization if possible:
    (a) Characterization by MALDI-TOF MS or other MS-based methods customized for oligosaccharide detection using neutral oligosaccharide standards of at least DP 3–10 (include the specific identity of the standards used for comparison).
    (b) Glycosyl linkage analysis using chemical derivatization with appropriate methods and standards.
    (c) 2D NMR spectra showing anomeric region correlations

(B) Minimum requirements for eliminating oligosaccharides as possible components of a given cranberry material. Any one of the following three recommendations would be acceptable.
  1. ^1^H-NMR spectrum (D_2_O) showing the absence of anomeric signals corresponding to oligosaccharides.
  2. UV and LCMS-based detection with negative results may be acceptable if oligosaccharide-appropriate derivatization and separation methods are applied.
  3. The application of a standard monosaccharide analysis method suitable for oligosaccharides and other complex carbohydrates yields 0% monosaccharides.

## 3. Human ADME of Cranberry Complex Carbohydrates

Complex carbohydrate phytoconstituents such as cellulose, hemicellulose, lignins, and polyphenolic compounds are often described as dietary fibers in studies investigating nutritional or general health effects. These compounds are classically considered to be high MW polymers and insoluble structural components, but the definition of dietary fiber has recently been modified to include lower MW, soluble oligosaccharides (DP 3–10 or 3–20) [107,110]. Various properties of prebiotic dietary fibers from multiple sources have been extensively and recently reviewed [76,97,98,110,111] and the potential prebiotic effects of cranberry oligosaccharides are discussed in Section 5.1.

Cranberry complex carbohydrates are likely to have similar absorption, distribution, metabolism, and excretion (ADME) profiles as other polymeric dietary fibers [97,110,158,159,160]. In the case of oligosaccharides, however, additional considerations may apply as several lines of evidence indicate that ingested dietary oligosaccharides can be absorbed from the GI tract and excreted into urine [158]. While oligosaccharides have been identified in human urine, the direct influence of plant-derived dietary oligosaccharides on human urinary oligosaccharide concentrations is not well understood [158]. The presence of cranberry oligosaccharides in human urine also has yet to be definitively confirmed. The general detection of soluble oligosaccharides in urine is complicated by the structural features and solution-state behaviors of these compounds (see Section 2.1.2) as well as the biological complexity of in vivo systems.

### 3.1. ADME of Classic Dietary Fibers

Most dietary fibers are too large (high MW) to be directly absorbed and therefore remain in the GI tract [110,159]. Humans lack digestive enzymes capable of degrading dietary fibers, but some types of fiber can be degraded by stomach acids [107,160]. Most prebiotic dietary fibers are initially degraded by gut microbiota using extracellular enzymes that can act on polymeric compounds [98,161,162]. The resulting oligosaccharide components may be acted upon by additional enzymes from other bacteria, and cross-species fermentation is typically involved in the degradation of structurally complex carbohydrate substrates [108,162,163]. Lower MW oligomers and monomers cleaved extracellularly are transported intracellularly using specific cell membrane transport proteins [164,165] where they may then be fermented to produce energy by various metabolic pathways (depending on the organism). Fermentation ultimately results in the production of short chain fatty acids (SCFAs) that may serve as substrates for other bacteria and for epithelial cells [110,111,166]. SCFAs may remain in the gut lumen where they modify the pH and bind to or chemically modify other molecules prior to excretion with the feces, or they may be absorbed into systemic circulation [109,166]. Circulating SCFAs may result in additional health benefits, and these compounds may eventually be excreted into either urine or back into feces [107,109,110].

Complex carbohydrate components that are not degraded by gut microbiota may be excreted unchanged in the feces but also contribute to beneficial health effects [98,107,109,110,111,166]. Polymers and oligomers contribute to fecal bulking, and the gel-forming and water-holding capacities of some fibers are believed to contribute to the formation of soft stools that are easy to pass [166,167]. Fibers may also associate with other molecules present in the gut lumen, including minerals, affecting their interactions with other compounds or substrates and carrying them through the GI tract until they are absorbed or excreted [158,166].

### 3.2. ADME of Soluble Oligosaccharides

The presence of a complex profile of neutral arabinoxyloglucan oligosaccharides in porcine urine after cranberry consumption indicates that these compounds can be absorbed from the GI tract, circulated systemically, and filtered from the blood by the kidneys to be excreted into urine after ingestion [24,25,26]. The similarity of compound elution profiles combined with the similarity of structures between porcine urinary oligosaccharides and cranberry oligosaccharides (Figure 1 and Figure 2) indicates that these compounds may be directly absorbed and excreted into urine without modification. This lack of metabolic modification would be consistent with their structural features, as the presence of a cellulose-type β-glucan backbone would prevent digestion by most human enzymes and would provide some chemical stability in the presence of digestive acids [158,160,167]. These observations for cranberry oligosaccharides are consistent with the findings of previous studies investigating urinary oligosaccharides from other dietary sources, as discussed below.

The presence of multiple types of oligosaccharides in mammalian urine from both dietary and other sources indicates that the kidneys are able to filter this class of compounds from blood into the urine [168,169,170,171,172,173,174,175,176,177,178,179,180,181]. Early studies using gel filtration, ion-exchange, and paper chromatography, combined with hydrolysis and chemical derivatization, characterized trisaccharides and oligosaccharides from healthy adult urine [168,169,170]. Studies of adult human urine have indicated the presence of high oligosaccharide concentrations associated with various endogenous disease states and have reported the presence of oligosaccharides in the urine of normal, healthy adults [171,172]. Infectious disease studies of adult humans have found unique oligosaccharide components in urine that correspond to those excreted by infecting *Schistosoma* parasitic worms [173], indicating that the kidneys are capable of filtering non-endogenous oligosaccharides from the blood. A limited subset of studies specifically investigating the ADME of dietary breast milk oligosaccharides in both human infants and piglets indicates that these compounds must be absorbed from the digestive tract before they can be circulated and excreted into urine [174,175,176,177,178,179,180]. Investigations using ^13^C labeled breast milk oligosaccharides indicate that urine contains oligosaccharides that are directly absorbed upon ingestion as well as those that may be derived from the degradation of breast milk polysaccharides by gut bacteria [176]. Animal models have also shown that oligosaccharides can be ingested and excreted into urine unchanged [178,180,181].

While studies investigating urinary oligosaccharides often hypothesize that endogenous oligosaccharides, such as those derived from glycoproteins, are the source of unknown urinary oligosaccharides [172], these compounds may also be derived from soluble dietary oligosaccharides that have been absorbed into circulation and filtered from the blood by the kidneys [158]. Dietary oligosaccharide uptake and distribution pathways that apply for mammalian infants and adult animals are also likely to apply for adult humans and for other dietary oligosaccharides, and yet little is known of such mechanisms or pathways in either infants or adults.

### 3.3. Detection and Analysis of Soluble Oligosaccharide Components In Vivo

Some of the unknowns regarding the presence and concentrations of dietary oligosaccharides in adult human urine may be attributed to the difficulty of isolating or detecting these compounds from in vivo systems [158,172]. Urinary oligosaccharides with prebiotic properties may be consumed by UT microbiota leading to overall reduced oligosaccharide concentrations prior to final excretion [97,166,182,183]. SCFAs produced as a result of urinary oligosaccharide fermentation may be consumed by both other bacteria and host epithelial cells. SCFA concentrations in urine may represent UT microbiota metabolites as well as circulated compounds derived from fiber fermentation by gut microbiota. During systemic circulation, oligosaccharides may associate with other blood components, including various cells, and the resulting complexes may be re-excreted into the feces [167,173,175,177,178,179,180]. Oligosaccharides that can be detected in or isolated from urine may therefore need to be present in excess of amounts required to saturate these and other in vivo dynamic interactions, and the use of relatively high doses of CHEP in the porcine urine study [24,25,26] likely facilitated the isolation and identification of cranberry oligosaccharide compounds in urine. Extensive ADME quantification studies that consider the above possibilities as well as others will therefore be necessary to investigate these compounds further.

The detection of soluble oligosaccharides in urine is further complicated by structural features and solution behaviors that require the application of specialized detection techniques such as those used previously for this chemical class [158,172,176,180,184]. Many modern, standard, high-throughput metabolomics techniques are currently unable to detect soluble neutral oligosaccharides (DP 3–10) in biofluids such as urine or plasma due to methodological and parameter limitations [91,92,172,185,186]. Such platforms are typically focused on small molecule (<1000 Da) isolation, and underivatized neutral oligosaccharides are invisible to detection methods such as UV and are poorly ionized in GC-MS or LC-ESI-MS [28,172]. Methods targeted to the enrichment and detection of specific oligosaccharides from biofluids, such as those that apply specialized MS techniques coupled to chemical derivatization and customized sample clean-up methods, are, however, capable of selectively characterizing a wide range of oligosaccharides with MWs from 500–3500 Da or higher [131,158,171,172]. Isotope ratio MS methods have also been successfully used to track the metabolism and distribution of ^13^C-labeled oligosaccharides [174,176]. Methods such as these may therefore facilitate future studies of plant-derived, neutral, soluble, dietary oligosaccharides in urine.

## 4. Biological Properties of Cranberry Complex Carbohydrates

Current evidence indicates that cranberry complex carbohydrates share many of the biological properties and beneficial health effects as dietary fibers from other sources [187,188,189]. These health effects go beyond “prebiotic” bioactivities, however, impacting microbiota by other mechanisms and directly affecting host health. Other dietary oligosaccharides have been found to affect bacterial adhesion, co-aggregation, biofilm formation, and growth responses with subsequent effects on the colonization of host cell surfaces [99,100,101,190,191,192,193]. Such effects would therefore impact the survival of an organism within a mixed bacterial community without serving as a growth substrate [99,100,101]. Various dietary oligosaccharides have also been shown to have direct effects on metabolic disease parameters and host immune responses that lead to host health benefits. These effects on overall host health subsequently impact microbial communities as a result of changes in host metabolism and cellular responses [188,189,194,195,196,197]. For example, the prevention of oxidation and inflammation-associated damage in the gut could protect epithelial cells from subsequent microbial invasion by promoting normal cellular function [197]. These types of biological properties have been observed in vitro for some of the cranberry oligosaccharide fractions discussed in Section 2.2 and Section 2.3, indicating that cranberry oligosaccharides may have similar effects in vivo as other dietary oligosaccharides.

### 4.1. Effects on Bacterial Adhesion

The inhibition of bacterial binding to surfaces is believed to be significant for human health as it prevents pathogenic bacteria from subsequently invading host cells [99,101,181,192,198,199]. Prevention of *E. coli* adhesion is of particular interest as this bacterium is commonly associated with symptomatic UTIs [198,199]. The prevention of adhesion has been found to be equally effective against both antibiotic-susceptible and antibiotic-resistant strains of *E. coli* [200] and adhesion prevention does not provide a selective pressure that can lead to resistance development [99,192].

Oligosaccharides from multiple dietary sources have anti-adhesion properties [192,193,201,202]. These include pectins, β-glucans, and xyloglucans with similar base structures as those of the cranberry oligosaccharides [77,190,191,192,193,202,203]. General mechanisms for oligosaccharide anti-adhesion activity include [99,192,203]: (1) direct interactions with bacterial adhesion receptors through adhesin mimicry, (2) indirect effects on bacterial adhesins through interactions with other structural components of fimbriae or cell surfaces, or (3) non-specific aggregation with bacteria by non-covalent binding to bacterial surfaces. Additional possible mechanisms may also apply, such as those involving effects on gene expression or organism interactions [99,152,192]. The mechanisms by which cranberry oligosaccharides exert their anti-adhesion effects on bacterial binding have yet to be fully elucidated, but are currently believed to be indirect or non-specific [26,112,204,205].

Multiple anti-adhesion assays have been used to assess the anti-adhesion properties of cranberry fractions containing oligosaccharides (Table 4). These include both human red blood cell (HRBC) agglutination and epithelial cell binding assays and have primarily focused on the use of uropathogenic P-fimbriated and Type 1 fimbriated *E. coli* strains (e.g., CFT073, MG1655), though additional *E. coli* strains have also been used. 

In vivo, bacteria such as *E. coli* may express multiple and diverse adhesion phenotypes that provide fitness advantages in specific environments [199,206]. Bacterial-epithelial cell adhesion assays are therefore more representative of the complexity of bacteria-host interactions within in vivo systems than those that depend on specific receptor binding such as bacterial-HRBC anti-agglutination assays [198,199,207]. The use of bacterial-epithelial cell adhesion assays enabled the early identification of the anti-adhesion properties of human urine after cranberry consumption [4,208], but the methods used were labor intensive and resource limited. Anti-agglutination assays based on the identified binding epitope of uropathogenic P-fimbriated *E. coli* [207] allowed for the use of more readily available HRBCs and could therefore be adapted for sample screening. These type of assays were subsequently used for cranberry studies and led to the identification of PACs as anti-adhesive constituents of cranberry materials [209,210,211,212,213]. Anti-agglutination assays also provided the first qualitative evidence that oligosaccharide-containing fractions lacking in polyphenolic compounds from both porcine urine (FA, HF, and HI fractions) and cranberry materials (CJA fractions) possessed anti-adhesion properties [24,25,26].

Improved quantitative anti-adhesion assays using human bladder epithelial cells (T-24: ATCC HTB-4) and either radiolabeled [214] or fluorescently labeled [215] P-fimbriated *E. coli* (CTF073: ATCC 700928) have since been developed. The labeling of *E. coli* facilitated the quantification of bacterial cells adhered to confluent layers of epithelial cells after a test sample treatment [214,215]. The radiolabeled version of this assay was used with a subset of WBP fractions (SNA oligosaccharides) and results indicated that oligosaccharide subfractions exhibited anti-adhesion properties and that mixtures of compounds may be required for effective anti-adhesion [28]. Subsequent use of the fluorescence version of the assay confirmed the anti-adhesion properties for oligosaccharide fractions from both CHEP (A6 fraction) [112] and CJS (CCA1 and CCA2 fractions) materials [25,29]. The CHEP material (A1) and the A6 oligosaccharide fraction [112] were further tested in additional anti-adhesion assays using T-24 epithelial cells with fluorescently labeled Type 1 fimbriated *E. coli* (UTI89) [215] and human colonic epithelial cells HT29 (ATCC HTB-38) with enterohemorrhagic *E. coli* (O157:H7, ATCC BAA-1883).

Both CHEP and the A6 xyloglucan fraction were found to inhibit bacterial adhesion to different relative levels [112]. The A6 fraction was found to have the greatest effect on the adhesion of type 1 fimbriated *E. coli* and was able to inhibit the adhesion of P-fimbriated *E. coli* in a concentration-dependent manner. Low concentrations (0.0001–0.1 mg/mL) of the A6 fraction were observed to block the binding of *E. coli* O157:H7 to colonic epithelial cells while higher concentrations were not able to inhibit adhesion. CHEP had the highest anti-adhesive effect on the adhesion of P-fimbriated *E. coli* to bladder epithelial cells but this effect was only slightly greater than that of the A6 fraction. As the A6 fraction contained all neutral oligosaccharide components present in the CHEP material, further interpretations could not be made regarding the relative contributions of different oligosaccharide components to the observed anti-adhesion effects.

The quantitative anti-adhesion results obtained for the CJS CCA2 oligosaccharide subfractions indicated that oligosaccharide-containing fractions could account for the majority of the anti-adhesion activity observed for the CCA material [29]. Although the CCA2 oligosaccharide subfractions contained distinct, closely related components (Figure 4 and Figure 9), all were observed to possess anti-adhesion properties, with the oligosaccharides that eluted the earliest from Sephadex LH-20 having the highest activity. Comparison of the CCA2 anti-adhesion results and elution and ^1^H-NMR profiles with previously obtained fractions (Figure 4 and Figure 9) also enabled us to further interpret the findings of our previous anti-adhesion studies for cranberry oligosaccharides [24,25,26,27,28,29].

The oligosaccharide fractions with the highest anti-adhesion activity in the three anti-adhesion assays used by our group included CJA1-02B, CJA2-02, CJA2-03, SNA-03, CCA1-01, and CCA2-01 [24,25,26,27,28,29]. These materials were obtained using similar methods and all contained different proportions of structurally related arabinoxyloglucan mixtures from three different cranberry source materials (CHEP, WBP, and CJS) (Figure 4 and Figure 9) [24,25,26,27,28]. Fraction SNA-02 was not tested in the anti-adhesion assay discussed in Auker et al. [28] and the significance of this omission was not recognized at the time. ^1^H-NMR data for SNA-02 were obtained in DMSO-*d*_6_, making direct comparisons to the data obtained in subsequent studies difficult, but comparison to ^1^H-NMR data for SNA-03 obtained in the same solvent and 2D NMR data (unpublished) made it possible to determine that SNA-02 also contained arabinoxyloglucan oligosaccharides [28]. Based on elution sequence, the SNA-02 fraction may be most similar in compound profile to the CCA1-01 and CCA2-01 fractions [29] and may therefore have contained arabinoxyloglucan constituents with higher relative activity than observed for SNA-03 and the purified cranberry oligosaccharides **2** and **3** (Figure 1) [28]. These cross study comparisons allow us to propose that the cranberry arabinoxyloglucan compounds with the highest relative anti-adhesion activity are those that elute earliest from Sephadex LH-20 in 70% ethanol [24,25,26,27,28,29], but isolation and full structural characterization of these subfraction components has yet to be performed.

### 4.2. Effects on Biofilm Formation and Bacterial Aggregation

Biofilms are significant for many human disease states, including dental caries, recurrent UTIs, and catheter-associated UTIs [216,217,218,219,220,221]. Biofilms formed by commensal communities of bacteria may also be important for promoting epithelial cell health by protecting epithelial cell surfaces from the adherence of harmful organisms and by other mechanisms [222,223,224,225,226]. Biofilm formation is a complex process that initially involves adhesion to a surface followed by changes in gene expression profiles to facilitate surface-based rather than planktonic growth [216,227]. These phenotypic changes involve the production of extracellular polysaccharides, specialized proteins, and enzymes that protect bacteria and facilitate nutrient acquisition [216,227,228]. The prevention of biofilm formation is preferred as a treatment option because extracellular polysaccharides make biofilms difficult to chemically remove from surfaces and can protect bacteria from antibiotics and other harmful environmental conditions [216,217,218,228]. In this regard, cranberry materials have been investigated for their ability to affect biofilm formation by a number of potential pathogens, and a range of effects and activities have been observed for different organisms and test environments (e.g., urine vs. saliva vs. juice) [63,229,230,231,232,233,234]. This range of responses by different organisms may lead to either beneficial or harmful effects on overall human health, depending on both the bacteria present and the host environmental variables involved.

Multiple mechanisms may be involved in the prevention or promotion of biofilm formation by compounds such as oligosaccharides. These include effects on bacterial adhesion to surfaces, bacterial aggregation, gene expression profiles, and nutrient uptake mechanisms, as well as other factors [216,218,227]. Additional effects that influence bacterial interactions, such as the nonspecific binding of interspecies signaling molecules or effects on interspecies co-aggregation, may also influence the formation of mixed species biofilms [218,219,227]. Assays intended to screen for the effects of different compounds on biofilm formation typically measure the amount of extracellular polysaccharide produced by the studied bacteria in the presence of a test sample with a particular surface type and set of culture conditions [217]. Multiple in vivo environmental variables may affect bacterial biofilm formation, however, such as the presence of urea in urine or total solute concentrations [63]. Both assays investigating the effects of cranberry oligosaccharide fractions on biofilm formation have measured the amount of biofilm matrix formed by individual bacterial species on an inert polystyrene surface [113,114]. One of these studies also investigated bacterial co-aggregation for two pairwise combinations of three bacterial species [113]. While the results of these studies are limited and must be considered preliminary with regards to the potential impact of oligosaccharides on in vivo biofilm formation, they do provide data for the development of testable hypotheses.

Sun et al. [114] investigated the effects of CHEP and it fractions on biofilm formation by nonpathogenic (MG1655) and uropathogenic, P-fimbriated (CFT073) *E. coli* strains, and observed concentration-dependent and strain-dependent results. The separation methods used by Sun et al. [114] were focused on removing possible phenolic components from the total oligosaccharide material and generated two phenolic-free oligosaccharide fractions, Cranf1b-F1 and Cranf1b-F2. Only one of these, Cranf1b-F2, was chemically characterized further as it was able to affect biofilm formation by both *E. coli* strains. The Cranf1b-F2 oligosaccharide subfraction contained higher arabinose content than previously reported for cranberry oligosaccharide fractions, and the unique characteristics of this subfraction (see Section 2.2.2) may have led to distinct effects on biofilm formation relative to other subfractions.

The contrasting biofilm formation profiles observed by Sun et al. [114] for the CFT073 and MG1655 *E. coli* strains indicate the type of complex, dynamic interactions that organisms may exhibit in the presence of different concentrations of cranberry material in vivo. Both the Cranf1W and Cranf1b materials had greater inhibitory effects on biofilm formation by the non-pathogenic MG1655 than the pathogenic CFT073. These measurements were taken at a single concentration value (1.25 mg/mL), however, and the true effects of each material on biofilm formation may be concentration dependent, as was subsequently observed for the enriched oligosaccharide subfraction Cranf1b-F2. Within a specific concentration range (0.156–1.25 mg/mL) Cranf1b-F2 promoted the formation of biofilm by the non-pathogenic *E. coli* strain MG1655, while inhibiting biofilm formation by the pathogenic *E. coli* CFT073 strain. This effect was reversed for the two strains at higher concentrations (2.5–10 mg/mL), with Cranf1b-F2 inhibiting the formation of biofilm by MG1655 to increasing degrees while minimally affecting CFT073 biofilm formation [114]. In vivo, these trends may indicate that the presence of cranberry oligosaccharides and other components at different concentrations may provide strain-specific and concentration-dependent competitive advantages in terms of host colonization and phenotypic profiles. These observations also indicate that in vitro comparisons using single concentrations for crude fractions may not accurately reflect complex, concentration-dependent effects. Additional in vivo considerations may further limit the possible interpretations of in vitro results, and additional studies are needed.

Neto et al. [113] assessed the ability of NDM, NDMEt, and NDMAc to inhibit bacterial co-aggregation using paired combinations of three different bacteria isolated from the human gingival crevice. An unspecified strain of *Fusobacterium nucleatum* (Fn) was mixed with a test sample and incubated prior to the addition of either *Streptococcus sanguinis* SS34 (Ss) or *Porphyromonas gingivalis* ATCC 33,277 (Pg). Bacterial co-aggregates were visually scored and the lowest sample concentration at which no co-aggregation occurred was reported as the MIC. A similar pattern of results for each fraction was observed for both pair-wise bacterial combinations (Fn+Ss and Fn+Pg) and indicated that the polyphenol constituents of the NDM material were the components primarily responsible for preventing bacterial co-aggregation. The authors [113] also observed that NDMEt was ineffective at preventing bacterial co-aggregation as it produced MIC values that were almost twice that of the positive control values. Though the authors [113] did not comment further, additional interpretations of these findings may also be relevant. The ability of bacteria to aggregate in the presence of relatively high concentrations of cranberry oligosaccharide mixtures may lead to beneficial effects in vivo for specific microbiota species or may facilitate the clearing of bacterial aggregates from urine or other solutions by unknown mechanisms. The mechanism by which co-aggregation occurs may also impact subsequent biofilm formation in vivo; the non-specific binding of bacteria into a soluble gel-like matrix composed of oligosaccharides (see Section 2.1.2) may prevent bacteria from adhering to surfaces and subsequently forming biofilm. Additional studies will be required to investigate the effects of oligosaccharides on the solution state aggregation of various bacterial species and subsequent adhesion and biofilm formation.

Neto et al. [113] also examined the relative abilities of NDM, NDMAc, and NDMAc-MCI subfractions to prevent in vitro biofilm formation of *Streptococcus mutans* (UA159), *Staphylococcus aureus* (MRSA), and *Staphylococcus epidermidis* (RP62A) in monoculture. For all three bacteria, NDMAc significantly inhibited biofilm formation at 0.08 mg/mL while NDM had significant effects at 0.32 mg/mL. In additional results for the inhibition of *Streptococcus mutans* biofilm formation, MIC values were reported as 0.5 mg/mL for NDM, >0.5 mg/mL for NDMEt, 0.25 mg/mL for NDMAc, and 0.125 mg/mL for NDMAc-MCI-3. The NDMAc-MCI-3 subfraction (30% *w*/*w* of NDMAc) contained both polyphenol and xyloglucan oligosaccharide components similar to those present in NDMEt, indicating that both chemical classes may contribute to the effects of cranberry materials on biofilm formation [113]. The authors concluded that “the observed anti-adhesion activities are due to a combination of epicatechin-containing polyphenols, flavonol derivatives, and oligosaccharides that are likely xyloglucans, and that these unique combinations of polyphenol and non-polyphenol constituents may act more specifically and effectively on some bacteria than others” [113]. These differences in specificity and efficacy may have consequences for mixed-species bacterial aggregation and biofilm formation in vivo that could result in microbiota profile changes that contribute to the health effects of cranberry consumption.

### 4.3. Effects on Microbial Growth and Cell Viability

Cranberry oligosaccharide and xyloglucan fractions and purified cranberry xyloglucan compounds have shown a distinct lack of antimicrobial properties or cytotoxic effects on numerous bacterial and human cell lines. Cranberry xyloglucan fractions have been screened by antimicrobial activity assays [25,28,132] and found to lack antimicrobial properties against various bacteria, fungi, and a few protozoa. Other authors have reported a lack of growth inhibitory effects as notes in the context of other assays [114]. Strains or related species of many microorganisms used for in vitro assays are commonly found within normal healthy microbiota populations. This reported lack of antibacterial and antifungal activity is therefore a positive result, as it indicates that oligosaccharides would not provide selective pressures or harmful effects for potentially beneficial organisms that are part of a normal, healthy microbiota [218].

In contrast to growth inhibitory effects, cranberry oligosaccharides may have growth promoting effects independent of the use of these compounds by bacteria as growth substrates. Sun et al. [133] reported that cranberry pectic oligosaccharide fractions of CHEP were able to promote the in vitro growth of quiescent and persister phenotypes of *E. coli* CFT073 under different culture conditions. Low concentrations of cranberry pectic oligosaccharide fractions Cranf1b-cPOS, HDP-cPOS, uG3^m2^, and uG4^m3^ (**8**, Figure 10) were able to induce bacterial growth when applied to glucose-deficient agar plates containing an overlay of non-growing (quiescent) *E. coli* [133]. The use of glucose-free media with similar methods indicated that the reversal of quiescence was not due to the use of pectic oligosaccharides as a carbon source, but rather to a stimulating effect from the presence of the pectic oligosaccharides that led to growth on glucose as the carbon source. These authors [133] also found that *E. coli* grown in the presence of both cranberry pectic oligosaccharides (Cranf1b-cPOS and Cranf1b-cPOS-t) and the cidal β-lactam antibiotic ampicillin, formed a significantly reduced quantity of persister cells. This result indicated that the presence of cranberry pectic oligosaccharides was able to induce the active growth and subsequent death of bacteria in the presence of the antibiotic. The molecular mechanisms for these apparent growth promoting effects of cranberry pectic oligosaccharides on *E. coli* are currently unknown but may lead to significant in vivo effects for *E. coli* survival in the presence of antibacterial drugs or other potentially harmful compounds. Growth promoting effects such as these may also apply for other bacteria with dormant phenotypes and may contribute to the overall effects of cranberry materials on biofilm formation and gut microbiota profiles.

### 4.4. Effects on Metabolic Health Factors

Cranberry materials have been attributed with antidiabetic, anti-inflammatory, anti-oxidant, and anticancer effects [1,2,3,7,12,13,14,15,16,17,235]. A small subset of in vitro and in vivo studies indicate that cranberry complex carbohydrates may contribute to these various reported biological properties and their associated health benefits through anti-oxidant, anti-inflammatory, and immunomodulatory mechanisms [113,115,143] independent of their effects on bacteria. These effects are consistent with those observed for human milk oligosaccharides [236,237], and may be additionally significant due to the likely presence of cranberry oligosaccharides in both systemic circulation and urine (see Section 3.2). Many of the general health benefits of cranberry materials with regards to metabolic diseases are consistent with those of oligosaccharides and other dietary fibers from various plant sources and interested readers are encouraged to consider the following reviews and studies [194,195,197,238,239,240].

The formation of advanced glycation end-products (AGEs) is a process known to be associated with the harmful effects of diabetes [15]. The oligosaccharide-enriched, phenolic-free Cranf1b-CL subfraction obtained by Sun et al. [115] inhibited the formation of AGEs in a concentration-dependent manner with a significantly higher degree of activity than the positive control, aminoguanidine. The Cranf1b-CL fraction showed superior DPPH (2,2-diphenyl-1-picrylhydrazyl) free radial scavenging activity (IC_50_ 0.68 mg/mL) than that of the positive control, the commercial anti-oxidant, butylated hydroxytoluene (IC_50_ 2.2 mg/mL). The methylglyoxal trapping capacity of Cranf1b-CL was also assessed to establish if the material was able to trap this reactive carbonyl species but the fraction was inactive, indicating that alternate mechanisms may be involved in the anti-glycation properties of the oligosaccharide fraction. The authors [115] proposed that metal chelating activities may be of significance and additional binding or interaction mechanisms may also be responsible.

Neto et al. [113] performed an oxidant scavenging assay using a clinical isolate of *E. coli* to assess how much active material became bound to bacterial cell surfaces. Assay results were based on the quenching of luminol-dependent chemiluminescence by compounds with anti-oxidant properties. They found that the NDM and NDMAc samples yielded results consistent with the total phenolic contents of these materials, supporting the role of phenolics for this type of anti-oxidant activity. In contrast, *E. coli* treated with NDMEt did not exhibit anti-oxidant activity in the assay, consistent with the low phenolic content reported for NDMEt. These results do not necessarily indicate that NDMEt did not bind to *E. coli*, but rather that it did not exhibit anti-oxidant properties detectable by the assay used. Cranberry oligosaccharide mixtures may instead exhibit anti-oxidant effects through alternate mechanisms as was observed by Sun et al. [115]. The overall anti-oxidant effects of cranberry materials may therefore be enhanced by associations between oligosaccharides and phenolic compounds or polyphenols, as the different classes of compounds may work by distinct mechanisms of action.

The pectic polysaccharide obtained by Popov et al. [143] from small cranberry (*V. oxycoccus*) exhibited anti-inflammatory and immunomodulatory activity in a mouse model of acetic acid-induced inflammatory colitis. Multiple measurements, taken on live animals and after the animals were sacrificed, indicated that the cranberry pectin (at 100 mg/kg) had a protective effect similar to that of prednisolone (at 5 mg/kg). Colonic myeloperoxidase activity was decreased in animals that consumed the pectin, indicating an inhibition of neutrophil accumulation in the colonic mucosa. This reduction in neutrophil levels was correlated to protective effects such as reduced epithelial cell disruptions and erosions [143]. Reduced levels of lipid peroxidation in the intestinal wall of cranberry-treated, colitis-induced animals indicated possible anti-oxidant properties. Investigation of mucous production indicated that cranberry-treated mice produced adherent mucous levels similar to those of healthy controls in spite of the oxidizing effects of the acetic acid treatment. In healthy animals, cranberry pectin showed no significant effects on colon mucosa secretion and adherence but did appear to decrease the inflammatory status of mice by reducing vascular permeability and slightly decreasing levels of peritoneal neutrophils and macrophages. These effects on peritoneal immune cells [143] are reminiscent of those observed by Bode et al. [236], who reported that milk oligosaccharides were able to exert anti-inflammatory action by binding to monocytes, lymphocytes and neutrophils and thereby preventing the binding of these immune cells to endothelial cells. Prebiotic effects (see Section 5.1) such as those observed for pectic oligosaccharides from other dietary sources [194] may also contribute to some of the findings of Popov et al. [143].

## 5. Cranberry Carbohydrates and Human Microbiota

In the past decade, numerous technological advances have improved our knowledge and understanding of the role that the human microbiota plays in supporting health or causing disease [241,242,243,244]. The Human Microbiome Project, with initial results published in 2007 [243], provided a significant foundation for this understanding, and research in this area has continued since its inception [222,245,246], leading to increasing recognition of the importance of dietary choices for human health [111,158,166,247]. The effects of diet [247,248,249], however, are only one of many possible variables that influence microbiome profiles; conventional, easily-observed demographic patterns, such as age, sex, or ethnicity, do not appear to affect microflora populations as strongly as other factors that are often not as easy to recognize or quantify. Human microbiota profiles have been correlated to genetics [250], host immune status [251], body site [252,253,254], and lifestyle choices [241,253,255]. Temporal fluctuations in microflora populations have been observed across hours to months, though the causes and mechanisms of such temporal effects are not yet well understood [241,253,256]. Although the possible significance of each variable has yet to be fully investigated, all of these factors likely correlate to microbiome diversity and fluctuations and all may impact the response of a given microbiome profile to a dietary intervention such as the inclusion of cranberry products.

While numerous studies have focused on profiling and investigating the gut microbiome, delayed recognition of the presence of the UT microbiota, with initial reports in 2012, has delayed our understanding of this microbiome [224,257,258,259,260]. Progress in UT microbiome research has also been hindered by the difficulty of accessing UT epithelial layers without the use of invasive procedures [225], the low microbial composition of urine samples [261], and the difficulty of culturing organisms that are primarily adapted to the extreme environment of the UT [258,259]. While the quantification of bacteria present in urine may not directly reflect the dynamic interactions of bacterial and UT epithelial cells in vivo [225], it does provide a starting point for additional research. The current understanding of the UT microbiota has been extensively reviewed by several authors [223,224,257,262], and a recent review has catalogued all microbes currently known to potentially inhabit the UT [263]. The UT microbiota represents 21.4% of the known prokaryotic diversity associated with humans and 62.3% of organisms identified in urine can also be found in the human gut microbiota [263]. As has been observed for the gut microbiome [239,243,244], current evidence suggests that microbiota profiles play significant roles in UT disease occurrence and progression [223,264,265,266,267,268], but extensive additional studies are needed to further elucidate complex host-microbiota interactions within the UT using methods and perspectives that have been applied to other microbiome studies [222,241,242,246]. The presence of soluble, plant-derived, dietary oligosaccharides in urine would be significant in terms of UT health, through both prebiotic effects on UT microbiota and direct biological and chemical effects, and the possible influence of these compounds must be considered in the context of both cranberry and other dietary sources.

Knowledge regarding the effects of cranberry materials and specific cranberry compounds on human microbiota is still minimal. For the few recent studies [74,75,150] that have investigated the effects of cranberry on in vivo gut microbiota profiles using 16S rRNA sequencing, interpretations of the results have been based primarily on a limited understanding of the composition of the cranberry materials used. Two studies used WBP prepared from freshly harvested cranberries [74,75], while a third used SDC [150]. All of these materials are likely to contain significant amounts of both oligosaccharides and higher DP cell wall polysaccharides and the findings of all three studies regarding microbiome shifts are consistent with the prebiotic effects of dietary fiber consumption. In spite of this, the authors of one of these studies [74] suggest that “cranberry constituents, yet to be fully identified, could exert their beneficial effects by helping maintain SCFA production in the gut of people consuming diets low in fiber and high in animal meats and simple sugars.” It may in fact be that the administered cranberry material provides the missing fiber component.

In the gut, cranberry oligosaccharides and higher MW dietary fibers are likely to be degraded and metabolized by pathways used by bacteria for the fermentation of similar compounds from other dietary sources [111,162,269,270]. Numerous investigations of other dietary oligosaccharides and complex carbohydrates [166,182,190,191,194,201,271,272,273,274] also report bioactivity and fermentation results consistent with those observed for cranberry oligosaccharides, specifically, and for whole cranberry materials that contain oligosaccharides and other complex carbohydrates. Consideration must therefore be given to the possible effects of cranberry oligosaccharides and other complex carbohydrates on gut and UT microbiota profiles and the resulting health benefits.

### 5.1. Prebiotic Effects of Cranberry Oligosaccharides

A number of current references discuss the known effects of various dietary oligosaccharides and other complex carbohydrates on both gut microbiota and human health [77,98,109,110,111,192,275]. Many polymeric and oligomeric dietary fibers are considered to be “prebiotic” as they selectively promote the growth of beneficial bacteria within the human microbiota [111,162]. The effects of prebiotic fibers on individual bacteria result in overall changes in microbiota profiles by a variety of mechanisms [161,165,276,277,278,279]. In general, the physiological consequences and health benefits of prebiotic fiber consumption are significant and diverse and the interested reader is referred to expert reviews for more information [98,107,109,110,111,196].

The unique abilities of different microbes to degrade complex oligosaccharides with specific structural features, such as those of xyloglucans, results in the selection for specific sub-populations of bacteria within a given ecological niche, leading to different types of prebiotic effects for different types of compounds [110,111,162,164,165,280,281,282]. Microorganisms may also work together to degrade specific carbohydrates with complex structures and the ability of a given microbial consortium to degrade a specific type of carbohydrate may depend on the presence and activity of multiple organisms capable of expressing different enzymes [111,166,280]. The presence of complex mixtures of carbohydrates may further result in diverse interaction-based effects for multiple organisms in vivo [166,282,283]. Microbial communities with different component members will also respond differently to the same compounds based on their overall metabolic capabilities [111,161,162,164,165], further compounding the potential health effects of a given prebiotic fiber.

Studies investigating the possible prebiotic effects of cranberry oligosaccharide fractions [131,132] have shown results consistent with the prebiotic effects for structurally related oligosaccharides with β-glucan backbones, including other xyloglucans, from other dietary sources [164,165,278,279]. While prebiotics are typically discussed in terms of the gut microbiota, cranberry oligosaccharides that are absorbed and excreted into urine may also have prebiotic effects on UT microbiota populations, leading to effects on overall UT health. Further studies are needed to characterize the prebiotic effects of cranberry oligosaccharides for both gut and UT microbiomes.

#### 5.1.1. Bacterial Fermentation of Cranberry Oligosaccharides in Monoculture

Ozcan et al. [131] investigated the ability of strains and subspecies of three bacterial species known to occur in the human gut to ferment cranberry xyloglucan components as a sole carbon source in vitro. The organisms used included three strains of *Bifidobacterium longum* ssp. *longum*, four strains of *Bifidobacterium longum* ssp. *infantis*, *Lactobacillus plantarum*, and *L. johnsonii*. Cranberry materials used included an enriched xyloglucan fraction (Cranf1b-XG) prepared by the authors based on previous studies [115] and the A2 oligosaccharide and A6 xyloglucan fractions previously prepared by Hotchkiss et al. [112].

Of the nine organisms tested [131], only *Bifidobacterium longum* spp. *longum* UCD401 and *Lactobacillus plantarum* ATCC BAA-793 were able to use the Cranf1b-XG mixture as a sole carbon source, and both were observed to ferment xyloglucans more slowly than glucose. *B. longum* spp. *longum* UCD401 produced a relatively modest amount of biomass as compared to growth on glucose, while the biomass produced by *L. plantarum* was comparable to that produced on glucose. Analysis of spent fermentation medium indicated a preference of *B. longum* spp. *longum* UCD401 for lower DP (<7) oligosaccharides, as higher DP molecules were found to accumulate. Comparisons of SCFA concentrations relative to those expected for glucose utilization indicated that *B. longum* spp. *longum* UCD401 employed metabolic adjustments to its usual pathway to more efficiently harvest energy from cranberry xyloglucans: levels of lactate decreased, acetate increased, and formate increased. The SCFA metabolic end products of *L. plantarum* fermentation indicated inefficient substrate utilization, as lactate production was decreased and acetate and formate production were increased relative to levels expected for glucose utilization. The authors [131] proposed that the reduced lactate concentrations combined with the increased production of acetate and formate by both organisms indicated that increased amounts of ATP were needed for the catabolism of xyloglucans.

When the A2 and A6 fractions [112] were tested for fermentation potential, more of the studied bacterial strains were able to grow, indicating that mixtures of compounds may have additive or synergistic effects on bacterial metabolism. The authors [131] also suggest that the different strains may have variable abilities to use oligosaccharides with different DPs and substitution patterns. The gene expression profile of *B. longum* spp. *longum* UCD401 on the A6 mixed composition xyloglucan fraction indicated the upregulation of genes involved in the fermentation and degradation of arabinose and xylose, including an α-L-arabinofuranosidase gene, two arabinan endo-1,5-α-L-arabinosidase genes, and a β-xylosidase gene. Genes involved in xyloglucan transport were also upregulated, including those for a carbohydrate ABC transporter substrate-binding protein and carbohydrate ABC transporter membrane proteins, indicating the probable uptake of arabinose and lower DP xyloglucans.

Results also indicated that mixed bacterial communities may have an increased capacity to ferment cranberry oligosaccharides, though the mechanisms of such interactions are likely to be complex [131]. Although *B. longum* spp. *infantis* ATCC 15,697 was unable to grow on Cranf1b-XG as a sole carbon source, this strain was able to grow when conditioned supernatants from *L. plantarum* or *L. johnsonii* grown on glucose were added to cultures. In contrast, spent supernatants from *B. longum* spp. *longum* UCD401 were not able to induce the growth of *B. longum* spp. *infantis* ATCC 15,697 on Cranf1b-XG. The growth rate of *L. plantarum* was increased in the presence of Cranf1b-XG when combined with spent supernatants from *B. longum* spp. *longum* UCD401 grown on Cranf1b-XG, but the amount of biomass produced was decreased, indicating possible competition for xyloglucan substrates or the presence of inhibitory substances.

Multiple possible cross-feeding and other interactions can occur between diverse bacteria in vivo, as additional gut microbes, including *Bacteroides* species and the Lachnospiraceae [111,164,165,280], also have enzymes appropriate for xyloglucan degradation, uptake, and fermentation. In addition, β-glucans from several dietary sources have been reported to enhance the growth of Lactobacilli in infant fecal microflora and to result in extended fermentation times [278]. β-Glucans from oats have also been shown to induce the growth of *Prevotella* and *Roseburia* with associated increases in propionate production [279]. Arabinoxyloglucan oligomers or polymers that are hydrolyzed by bacterial enzymes to form β-glucans or other related compounds may therefore serve as substrates for additional bacterial species. Further detailed studies are necessary to characterize the abilities of other gut microbes to use cranberry complex carbohydrates as a carbon source, individually and in mixed culture, and to identify and investigate the mechanisms of possible in vivo interactions.

#### 5.1.2. Bacterial Fermentation of Cranberry Oligosaccharides in Mixed Species Culture

The presence of different ratios of specific cranberry components in different products may lead to different types of effects on different starting microbiota profiles. The results of a study using an in vitro gut simulator model and two donor microbiota populations [132] indicate that WBP, phenolic-enriched, and oligosaccharide-enriched cranberry fractions may lead to distinct effects on the growth of the five most abundant bacterial families examined (Bacteroidaceae, Ruminococcaceae, Lachnospiraceae, Enterobacteriaceae, and Porphyromonadaceae). The data included a wide standard error range for sample replicates, but trends indicated that the effects of WBP may be greater than, and sometimes the opposite of those observed for the separated oligosaccharide-enriched and phenolic-enriched fractions [132]. For example, in donor microbiota lacking in Enterobacteriaceae, the relative abundance of Bacteroidaceae (Bacteroidetes) was increased by as much as three times the control level when cranberry WBP or either fraction was present. In contrast, the relative abundance of Lachnospiraceae (Firmicutes) decreased when WBP was present, but increased (similar to the control) when either fraction was present. In donor microbiota with a higher (15%) than usual (0.1–1%) relative abundance of the Enterobacteriaceae (Proteobacteria), the relative abundance of this family was decreased in the presence of WBP or the oligosaccharide-enriched fraction (similar to the control), but was increased in the presence of the phenolic-enriched fraction. Increased abundance was observed for the Bacteroidaceae and Porphyromonadaceae families (Bacteroidetes) in the presence of the WBP or oligosaccharide-enriched fraction, with minimal increases in these families resulting from the presence of the phenolic-enriched fraction. These results suggest that the different components of phenolic-enriched and oligosaccharide-enriched fractions may have different effects on microflora populations, and that the combined effects of the two fractions are complex [132].

While the authors [132] discussed the possible general effects of cranberry materials on gut microbiota populations, similar direct and differential effects may also occur for UT microbiota populations depending on the subset of cranberry components and related metabolites that end up in urine. The families Ruminococcaceae and Lacnhospiraceae are both members of the phylum Firmicutes, while the Bacteroidaceae and Porphyromonodaceae are both members of the phylum Bacteroidetes. The Firmicutes and Bacteroidetes phyla are believed to make up about 30 and 6% of the UT microbiome, respectively, while these numbers are higher for the gut microbiome (50 and 10%, respectively) [263]. The phylum Proteobacteria makes up about 36% of the UT microbiome (in contrast to 19% of the gut microbiome) and contains the family Enterobacteriaceae [263]. While *E. coli* and other bacteria believed to be uropathogens are within the Proteobacteria and Enterobacteriaceae classifications, organisms within this group may also be significant as colonizers of a healthy UT [263]. The observations of O’Connor et al. [132] provide an interesting starting point for further investigations of the differential effects of cranberry materials on human microbiota, but specific interpretations of the study results are limited by a lack of genus and species level identification and minimal chemical characterization of the cranberry fractions used (see Section 2.2.2).

### 5.2. Effects of Cranberry on Gut Microbiota Profiles In Vivo

**Figure 11:**
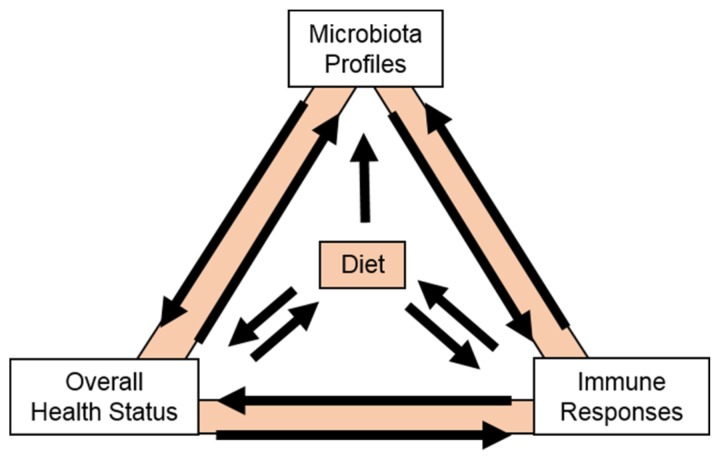
Depiction of the complex interactions that can occur as a consequence of a dietary intervention. Dietary choices are influenced by overall health status and immune responses and can, in turn, affect these parameters independently and through effects on the microbiota.

The results of in vivo studies may be influenced by the composition of the cranberry material used, the composition of the starting microbiota, and the overall health status of the individual (Figure 11). In vivo, the prebiotic effects of oligosaccharides (see Section 5.1) and other cranberry carbohydrates may combine with their biological and chemical effects (see Section 4), as well as with the effects of multiple other compounds. The complex and dynamic effects of multiple compound and organism interactions may result in microbiota profile changes that correlate to a range of health benefits. Detailed aspects of each of these interdependent factors further impact microbiome profiles, immune status, and overall health status for a given individual, leading to reciprocal and iterative responses to a given set of treatment conditions on a range of time scales. Ultimately, a given microbiome profile may be both a cause and a consequence of a given intervention, and these type of complex, dynamic interactions would lead to highly individualized responses to cranberry ingestion.

For multiple in vivo studies, cranberry consumption has been associated with minimal effects on measured health parameters for relatively healthy individuals [74,75,143,284]. These minimal observed effects suggests subtle changes in microbiome profiles, or direct effects on host cells and immune function [285], that may not be apparent without significant perturbation or intentional investigation. The direct effects of cranberry consumption may therefore be primarily detectable under stressed or disease-inducing conditions [74,284] or when specific methods are employed to track individual effects [286,287]. The use of animal models, where diseased states can be induced [75,143] or genetically selected [288], allow for more controlled and extreme conditions than typically occur in humans [289], and has provided a starting point for understanding possible mechanisms for the health effects of cranberry materials. Relative to animals with extreme versions of disease conditions, however, the response to cranberry administration for an individual with a normal health status may be similar in nature but lower in magnitude and therefore more difficult to detect. A similar pattern of response, with minimal detectable results for generally healthy individuals, has been observed for other investigations of dietary fibers [167]. Study designs must therefore consider the possible properties of the bioactive compounds being studied within the specific context of the unique health status parameters for each patient population being investigated.

Initial host microbiota populations are known to influence individual responses to dietary changes [247,248,249]. The normal diet pattern of an individual dictates their initial gut microbiome profile and the metabolic and biosynthetic potential of that microbiome [290,291,292]. In a study of the effects of SDC consumption on microbiome profiles [150], the observed difference in the microbiome abundance results for the single vegetarian consumer, relative to that of the other nine omnivore study participants, is consistent with the differential effects observed for other studies of fiber consumption for vegetarians versus omnivores [165,292]. Differences in initial microbiome profiles have also been correlated to differential responses to different fractions of cranberry materials [133], indicating that individuals with distinct initial microbiome profiles may respond in highly individualized ways to cranberry consumption.

Simplified, standardized diets for animal and human in vivo studies have been used in an effort to remove some of these variables in terms of biosynthetic potential, intentionally selecting for a dominant microbiome abundance profile in order to detect the effects of an added dietary component that is distinct from the composition of the standardized diet [248]. This type of approach has been successfully applied for an in vivo investigation of the effect of cranberry consumption when healthy adult humans were fed a consistent regimen of a high-protein, animal-based diet composed of meat, dairy products, and simple carbohydrates [74]. While changes in the relative abundance of individual microbiome groups as a result of a dietary intervention may be significant, the ultimate impact of such changes on human health parameters and various disease states is still an open question [246,260].

For microbiome profiling studies of cranberry materials, the level of microbiome classification and the methods of analysis used may have a significant impact on our ability to interpret the results. For a mouse in vivo model with induced colitis [75], the addition of cranberry to the mouse diet effectively reversed some of the diversity-decreasing effects of colitis, but the diversity changes were subtle. Changes weren’t significantly apparent at the phylum level but were readily observable at the family and genus levels [75]. Changes in phylum-level diversity have been observed to correlate to human SDC consumption [150] but this study did not include genus and species level identifications, limiting possible interpretations of the results. For the human study investigating the effects of cranberry addition to an animal-based diet [74], overall microbial diversity parameters were not significantly affected by dietary treatments, but changes in the relative composition of microbial populations were observed at multiple levels of classification. In this case [74], the negative effects of the animal-based diet on microbiota composition at lower levels of classification were reversed by the addition of WBP to the extent that there were no significant differences between pre- and post-diet samples when cranberry was included.

While it is tempting to compare and generalize the results of these studies and apply them to larger populations of individuals, this must be done cautiously. Together, these initial microbiome profiling studies [74,75,150] indicate that the consumption of cranberry materials may beneficially affect microbiome profiles and offset some of the negative effects of inflammation, promoting the abundance of the phylum Bacteroidetes and several genera of organisms believed to be beneficial for overall health. Unknowns far outweigh the knowns, however, in terms of how these findings may change with different study populations, in the presence of various diseases, or as a result of diverse dietary components or host factors [293]. The applicability of these findings to other cranberry materials is also unknown, but the diverse chemical composition of multiple products is likely to have significant and differing effects on possible results (see Section 1).

Multiple compounds found in cranberry are known to have a variety of effects on different microbes. For example, organic acids, polyphenols, flavonoids, and complex carbohydrates are all known to have different effects on microbial growth, adhesion, and biofilm formation [3,7,99,193,202,203,204,205,212,294,295,296,297,298,299]. These effects would combine in different ways to influence overall microbiome profiles in vivo. Observed results would be dependent upon initial microbiome profiles and many variables, including the relative concentrations of the compounds present, compound bioavailability, food matrix effects, and other factors [125,127,128,283,287,300]. Human and microbial metabolites of cranberry compounds [301,302] further expand this variability and the production of such metabolites would also be dependent on multiple host and microbiome factors. Combinations of biological, chemical, and prebiotic effects for individual compounds and their metabolites adds further complexity to the ways in which different compound mixtures may influence the survival of individual microbes in vivo [100,196]. Changes in organism interactions resulting from differential responses to different compounds may lead to reciprocal and iterative biological effects, further complicating possible interpretations [245,286,303,304]. To attribute the effects of cranberry materials on microbiome profiles to any one class of compounds (such as only the polyphenols or only the oligosaccharides) would therefore be an overly simplified and grossly inaccurate conclusion, as all components likely contribute in unique ways to a given microbiome response and an ultimate health effect for an individual.

### 5.3. Effects of Cranberry on Urinary Tract Microbiota Profiles

The discovery that the UT is not inherently sterile [259,305] changes everything about how UT health and UT “infections” must be studied and considered; we must redefine and re-conceptualize how we think of “infection,” “prevention,” and “health” [306]. Methods for the diagnosis of UT diseases must be reconsidered, as the simple presence or absence of *E. coli* detected in urine, using highly selective classical culture methods, is unlikely to sufficiently represent an individual patient’s UT health status [225,259]. The UT contains its own ecosystem of bacteria interacting with host cells and host systems in a chemically complex and constantly dynamic environment, much like the gut but with an additional, unique set of environmental parameters that may affect microbial survival. The “prevention of infection” may therefore be better defined as the prevention of symptomatic disease by modifying the presence and activities of specific microbes within the UT microbiome that promote health or prevent harmful effects or imbalances within an existing population of microbes.

This new recognition of the UT microbiota and its significance for UT health versus disease indicates that studies intending to investigate the ability of cranberry materials to prevent UTIs may actually have been unknowingly investigating the effects of cranberry consumption on UT microbiota populations. This perspective, when combined with diverse host factors and a limited understanding of cranberry material chemistry, readily explains the high degree of inter-individual variability in response to cranberry product administration [4,73,208,213] and the resulting controversy over cranberry clinical trial results [19,20,21,22,23]. To further add to this, the composition and solute concentration of urine is highly dynamic in response to overall health and diet [185], and may be further influenced by the metabolism of UT microbiota populations. Owing to the presence of a primarily liquid environment (urine) beyond the surfaces of host cells, adaptations such as adhesion and biofilm formation would be significant for the survival of both beneficial and harmful microbes within the UT [260,307]. The morphology, physiology, and surface chemistry of UT epithelial cells also differs from that of gut epithelial cells, further influencing the ability of bacteria to adhere. In addition to these and other UT related consideration, all of the factors and variables discussed for the effects of cranberry constituents on gut microbiota profiles (see Section 5.2) may apply for the UT microbiome.

The presence of dietary oligosaccharides in urine gains new significance from the perspective of the UT microbiota and inspires the proposal of many new hypotheses for how these compounds may affect UT health in vivo. As discussed in this review, in vitro support is already available for some of these hypotheses. Oligosaccharides may differentially affect the bacterial adhesion and biofilm formation of beneficial and harmful microbes, contributing to the ability of human urine to prevent the adhesion of potential uropathogens [4,208]. Generalized binding to bacterial surfaces and co-aggregation in solution may facilitate the clearing of bacteria in suspension through urination and flushing. Beneficial microbes in the UT may be able to use oligosaccharides as growth substrates, leading to SCFA production and changes in abundance profiles within the UT microbiome that affect inflammation and disease symptoms. Chemically, the formation of oligosaccharide aggregates may lead to the binding of other compounds in solution, limiting or increasing their bioavailability, and contributing to anti-oxidant and other protective effects against harmful substances. Oligosaccharides may also associate with surface carbohydrates and mucin layers on host epithelial cells to provide protective effects and generally prevent cellular invasion by harmful bacteria. Combined effects of oligosaccharides with other compounds and metabolites from cranberry as well as various urinary compounds may lead to further complex, dynamic, and reciprocal effects on UT microbiota.

Investigation of these and other proposed hypotheses will require recognition and consideration of the UT microbiota as a significant aspect of any future in vivo study of cranberry materials. Consideration must also be given to any effect that cranberry administration has on overall host health that could be reflected in urine composition. As cranberry product composition may directly affect both urine composition and overall health effects, our knowledge regarding the chemical composition of diverse cranberry products must be improved. True understanding of the effects of cranberry as a dietary intervention for promoting UT health and “preventing UTIs” may ultimately require approaches that incorporate concepts of both systems biology and personalized medicine [286,287].

## 6. Cranberry for Human Health and Disease Prevention

### 6.1. Recognized Composition Influences Bioactivity Interpretations

The hidden occurrence of soluble, neutral oligosaccharides in a wide range of cranberry products casts doubt on the biological properties of mixed composition cranberry materials that have been previously attributed to polyphenols. The limited descriptions and origins given for various cranberry materials and the confusion that exists with regards to material equivalency (see Section 1.1., Section 1.2., and Section 2.4) has also confounded the possible interpretations of multiple studies investigating the biological effects of cranberry products. Unless the material used in any given study was fully purified and structurally characterized by NMR spectroscopy, it must be assumed that multiple components beyond those discussed by the researchers could be contributing to the observed biological effects, including oligosaccharides and other compounds. In spite of this, many studies continue to attribute observed biological effects primarily to polyphenols, flavonoids, and other phenolic compounds [132,149,155,288,308]. Some studies have even directly reported carbohydrate contents as high as 95% for their materials and yet still discuss their results in the context of the more readily detectable and more frequently characterized anthocyanins, flavonols, organic acids, and PACs [308]. This type of bias towards flavonoids and polyphenolic compounds as the primary significant biological components of cranberries may predispose other researchers to avoid considering additional possible explanations for their results and further delay overall progress for future investigations of cranberry bioactivity.

For example, one result of this type of bias is that cranberry complex carbohydrates are likely to be unrecognized but significant contributors to the immune-related and cardiometabolic health benefits associated with cranberry materials [3,7,14,17,309]. The fermentation of cranberry complex carbohydrates may lead to beneficial microbiota profiles and SCFA production [74,131,132] that, together, promote epithelial cell health and cholesterol lowering effects as has been observed for other dietary fibers [165,187,189,303]. The results of several in vivo cranberry studies using mouse models [75,143,288] are consistent with the anti-inflammatory and immunomodulatory activities of pectin and pectic oligosaccharides from other dietary sources [194,285], and pectins from other foods have been associated with cholesterol lowering effects [238,303]. The anti-oxidant properties of oligosaccharides [115] may contribute to the overall anti-oxidant effects of cranberry materials through mechanisms distinct from those of polyphenols [235]. The aggregation and gel forming properties of multiple complex carbohydrates from cranberry may support the health and function of the epithelial mucous layer [310] by physically binding to mucins and providing a protective gel layer, as has been observed for tamarind xyloglucans [311,312]. The reduction in secondary bile acid levels as a result of cranberry consumption in the presence of an animal-based diet [74] may relate to the ability of various cranberry components to bind to bile acids [126]. Decreases in bile acid levels have been associated with the cholesterol-lowering effects of certain foods, such as oats, and with the detoxification of harmful and carcinogenic metabolites [126,187,189,304]. Investigations of other dietary fibers have indicated that fiber viscosity may influence bile acid binding and subsequent cholesterol lowering effects [189] and the high viscosity of soluble cranberry pectins [139] may therefore contribute to the bile acid binding and cholesterol lowering effects observed for cranberry materials [7,154,309].

Many of the general properties of oligosaccharides and other complex carbohydrates have yet to be specifically investigated for cranberry compounds, but even these few examples show the wide range of possible ways in which cranberry complex carbohydrates may influence the reciprocal interactions between microbiota populations and immune responses in vivo. These interactions may subsequently lead to cardiometabolic health effects by a variety of mechanisms. Ultimately, the dynamics of these interactions are complex and poorly understood and multiple types of studies will be required to further elucidate possible mechanisms for the overall health benefits of cranberry materials.

Oligosaccharides may also be unrecognized contributors to many of the oral health benefits associated with cranberry materials. Multiple researchers in the field of oral health have worked with cranberry NDM over nearly three decades from 1989–2017 [5] and yet oligosaccharides had not been previously characterized as possible components. The confirmed identification of oligosaccharides as a significant component (~70% *w*/*w*) of previously described NDM [113] is a strong example of the possible significance of the hidden occurrence of oligosaccharides. Most studies of NDM focused on PAC content as the cause of observed bioactivity but this emphasis must now be reconsidered for all studies using NDM prior to 2017. While PACs undoubtedly have numerous biological properties, consideration must also be given to the possible contributions of oligosaccharide components to the effects observed for NDM. Combination effects are also possible, as both oligosaccharides and polyphenols are known to non-covalently associate with a wide range of molecules, and concentration-dependent aggregation behavior in solution may influence observed bioactivities.

It is not possible to review all studies that might be associated with the potential biological effects of cranberry oligosaccharides, as many have not recognized the possible contributions of these compounds to the observed results. Because of the unrecognized presence and significance of oligosaccharides, however, new questions must be considered with regards to all previous cranberry research on mixed composition cranberry materials. Consideration must be given to whether the observed biological effect or health benefit could be due to oligosaccharides or to some other unrecognized component rather than to just polyphenols or other previously identified constituents. The exact contributions of different complex carbohydrates to the biological properties previously attributed to various cranberry products will require focused experiments and detailed chemical examinations of the materials used. Investigators will also need to consider oligosaccharides and other complex carbohydrates as possible significant contributors to the biological activities reported for mixed composition cranberry materials in both past and future studies.

### 6.2. A New Paradigm for UTI Prevention with Cranberry Materials

The conventional clinical paradigm regarding the use of cranberry materials for UTI prevention is based on the presence of components in human urine as a result of cranberry consumption that prevent infection by preventing *E. coli* adhesion to uroepithelial cells [4,23,313]. The compounds most often cited as being responsible for the observed anti-adhesive properties of urine are PACs [314] in spite of the current understanding that these compounds are unlikely to be directly excreted into human urine at significant concentrations, if at all [315]. Current and recently published clinical trials continue to focus on PACs as the primary bioactive constituents that lead to the prevention of UTIs [156,316], in spite of the diverse chemical composition of the products being used and the high probability that oligosaccharides are a significant component of that chemical diversity.

As described in this review, we now know that oligosaccharides are present in many cranberry products, including juices, and may constitute a significant portion (~20% *w*/*w*) of most materials described as “cranberry powder” or “cranberry extract powder,” including some of the capsule-based materials used in clinical studies. Highly enriched cranberry oligosaccharide fractions (no phenolics) have been shown to affect the adhesion and biofilm formation of both beneficial and pathogenic microbes. Oligosaccharide fractions have been observed to have prebiotic effects on microbial growth in vitro with individual organisms and using in vitro gut simulators containing human gut microbiota populations. Whole cranberry materials have been used in vivo in both mice and humans for studies of gut microbiome profile changes and results are consistent with the presence of prebiotic and biologically active dietary fibers. Multiple general health benefits attributed to cranberry products are consistent with those observed for other prebiotic oligosaccharides and diets containing high fiber content. Oligosaccharides structurally related to those found in cranberry materials have been detected in and isolated from the anti-adhesive fractions of porcine urine, indicating the urinary bioavailability of these compounds, consistent with the urinary bioavailability of other dietary oligosaccharides in humans. Both the entire oligosaccharide mixture and subfractions containing discrete groups of oligosaccharides have demonstrated in vitro abilities to inhibit *E. coli* adhesion to uroepithelial cells. Additional properties have been observed for oligosaccharide mixtures in vitro that suggest additional biological and chemical properties in vivo.

The recently recognized importance of the human UT microbiota changes the paradigm for studies investigating any aspect of UT health or disease, and has significant implications for all clinical trials investigating the ability of cranberry products to support UT health or prevent UTIs. Future clinical trials must consider the effects of multiple cranberry constituents on beneficial and harmful microbes, and on overall host health parameters that may influence these microbes. Endpoints must account for the presence and function of the UT microbiota, eliminating the use of outdated classical clinical culture methods [259] and considering more relevant and quantifiable descriptors of patient health responses. The importance of in vivo studies for assessing the complex, dynamic effects of mixed cranberry materials on health is highlighted by the results of a recent study that suggest cranberry compounds may influence urinary excretion levels of uromodulin [316], a protein that is expressed in the kidneys and is one of the most abundant proteins in normal human urine [317]. The function of this urinary protein is not well understood, but it is associated with both cardiovascular and UT health and may influence overall UT host-microbiota interactions [317,318]. Trials investigating catheter-associated UTIs [319,320] already typically consider the unique microbiota profiles associated with these devices, but must now also consider the impact that cranberry materials may have on both initial patient UT microbiomes and on the development of both catheter biofilms and subsequent UTIs [63,321]. Clinical trials that investigate the concurrent use of cranberry materials and probiotics [313] must consider the possible prebiotic effects of cranberry oligosaccharides on both the existing microbiota and the provided probiotic organisms, as well as the possible colonization of the UT with the probiotic organisms [322,323].

While cranberry oligosaccharide fractions may provide us with a significant new tool for focused investigations of cranberry bioactivity, the use of refined cranberry oligosaccharide constituents in vivo may, however, be both impractical and ineffective. Oligosaccharides are only one class of numerous cranberry constituents that may affect both host systems and the human microbiome, resulting in diverse and compounded effects on overall health. Product cost must also be considered as a significant factor for products that are to be regularly consumed by patients over long term administration periods [324]. Materials such as WBP, CHEP, CJS, CJS-derived juices, and other conventional food products (Figure 5), with reduced refinement costs, may therefore be the most appropriate administration forms until more is understood about the various mechanisms by which different cranberry components work together to influence overall health.

In summation, the new paradigm for cranberry bioactivity is that oligosaccharides and other complex carbohydrates are significant components of cranberry materials that contribute to cranberry bioactivity through multiple mechanisms affecting both the host and resident gastrointestinal and urinary tract microbiota, thereby promoting beneficial host-microbe interactions and preventing disease symptoms.

## 7. Conclusions

The process of scientific discovery is never simple, especially for complex biological systems with seemingly innumerable variables. A physician’s ability to diagnose, treat, and prevent a given disease relies directly on our current level of understanding of that disease state and the mechanisms of action for the treatments being used. As our understanding is constantly evolving, often by leaps and bounds as well as through steady progression, it can be difficult for a single physician or researcher to stay abreast of significant new developments in multiple specific research areas. Sometimes, cross-disciplinary developments emerge, or discoveries made in a seemingly unrelated field become significant. When this happens, it becomes even more difficult for researchers and physicians to stay abreast of current knowledge and consider all the factors that may influence their experimental designs or treatment recommendations. While focused, detailed efforts are necessary for steady progress, it is also important to occasionally view the broader picture to be sure that this steady progress is investigating relevant details. Our hope is that this review of cranberry soluble oligosaccharides and other complex carbohydrates will provide a significant new perspective that can help the field of cranberry research progress in a new direction.

## Figures and Tables

**Table 1:** Descriptions of cranberry materials with suggested abbreviations.

Description	Abbreviation
Cranberry Hull Extract Powder (“Juice” Powder)	CHEP
Cranberry Juice (fresh pressed or from CJS, no additives)	CJ
Cranberry Juice Blend (with added fruit juices) – from CJS	CJB
Cranberry Juice Cocktail (with added sugar) – from CJS	CJK
Cranberry Juice Syrup/Concentrated Juice Syrup	CJS
Cranberry Pomace (fresh pressed or frozen)	CP
Cranberry Sauce (Whole: WCS/Jellied: JCS)	CS
Dried Cranberry Pomace – from CP)	DCP
Fresh Whole Berries	FWB
Sweetened Dried Cranberries – from FWB	SDC
Whole Berry Powder – from FWB	WBP

**Table 2:** Oligosaccharide-containing fraction names, sources and analyses for initial studies employing solvent/solvent partitioning and Sephadex LH-20 chromatography with 70% ethanol.

Starting Material	Study	Oligosaccharide Fractions	Chemical and Spectroscopic Analyses	Bioassay Testing
CHEP	[24,25,26,27,28]	CJA1, CJA1-02, CJA1-02B, CJA1-03B, CJA2, CJA2-02, CJA2-03, CJA2-04, CJA2-05 Pure compounds **2** and **3**	NMR (1D, 2D) PDAD, ELSD Glycosyl Composition Glycosyl Linkage Glycosyl Configuration	Bacterial HRBC Anti-Agglutination Bacterial Anti-Adhesion Microbial Growth Inhibition
WBP	[27,28]	SNA, SNA-02, SNA-03, SNA-04 Pure compounds **2** and **3**	NMR (1D, 2D) PDAD, ELSD Glycosyl Composition Glycosyl Linkage Glycosyl Configuration	Bacterial Anti-Adhesion Cell Viability/Cytotoxicity
CJS	[24,29]	CCA1, CCA1-01, CCA1-02, CCA1-03, CCA2, CCA2-01, CCA2-02, CCA2-03, CCA2-04, CCA2-05	^1^H-NMR PDAD, ELSD	Bacterial Anti-Adhesion

**Table 3:** Oligosaccharide-containing fraction names, sources, and analyses for targeted studies employing various separation methods.

Study	Oligosaccharides Fractions	Separation Sorbent: Elution Solvent	Detection/Fraction Partitioning Basis	Chemical and Spectroscopic Analyses
[72,112]	A1 (CHEP) A2 A6	SNAP KP-C18-HS: water, 15% MeOH, MeOH Sephadex LH-20: water CarboPrep 90 (PGC): 30% MeCN/0.1% TFA 2x TSK GMPW_XL_: 0.05 M NaNO_3_/0.01% NaN_3_	volume/solvent/time MALLS-DPV-RI (TSK gel)	NMR (1D, 2D); UV (230 & 280 nm) Neutral sugar content Uronic acid content Degree of methyl esterificationDegree of acetylation Glycosyl Composition MALDI-TOF/TOF MS/MS (CID)
[114]	Cranf1 (CHEP) Cranf1b Cranf1b-F1 Cranf1b-F2	SNAP KP-C18-HS: water, 15% MeOH, MeOH Sepharose Q XL 16/10: 0.1 M NaCl Bio-gel P2: water TSK gel G3000PW: water	Phenol sulfuric acid assay [138] for total carb content Refractive Index (TSK gel)	HP-SEC-RI for MW Glycosyl Composition Glycosyl Linkage NMR (1D, 2D); MALDI-TOF-MS
[115]	Cranf1 (CHEP) Cranf1b Cranf1bA Cranf1b-CL	RediSep GOLD C18: water, 15% MeOH, MeOH Hypersep Hypercarbon (PGC) SPE: 30% MeCN/0.1% TFA C18 RP SPE (not specified): water	volume/solvent/time	Glycosyl Composition ^1^H-NMR MALDI-TOF-MS
[131]	Cranf1 (CHEP) Cranf1b Cranf1b-XG	same methods as [115] to obtain Cranf1b Sephacryl S-100 HR 16/60: water	Phenol sulfuric acid assay [138] for total carb content	^1^H-NMR MALDI-TOF MS
[133]	Cranf1 (CHEP) Cranf1b Cranf1bA Cranf1bS-cPOSt Cranf1b-cPOSHDP-cPOS uG3m2 uG4m3 (pure 8)	same methods as [115] to obtain Cranf1b Hypersep Hypercarbon (PGC) SPE: water, 10% MeCN/0.1% TFA, 30% MeCN/0.1% TFA Trituration with 95% EtOH TSK gel Amide-80 HR HILIC	volume/solvent/time ESI-MS profile (TSK gel)	LC-ESI-MS/MS HR-ESI-TOF-MS/MS UV (235 nm) NMR (1D, 2D) Glycosyl Composition Uronic Acid Composition
[113]	NDM (from CJS) NDMEt NDMAc NDMAc-MCI-3	Dialysis membrane (12–14 kDa): water Dialysis membrane (12–14 kDa): 50% EtOH Sephadex LH-20: 50% EtOH, 75% Acetone MCI Gel CHP20P: 30, 50, 70, 100% MeOH	physical partitioning volume/solvent/time	Total Phenolic Content (Folin Ciocalteu) HPLC-PDAD (210–600 nm) ^1^H-NMRMALDI-TOF MS (DHBA)

See the Supplementary Materials Table for additional details regarding each fraction and for a list of abbreviations used in this table and elsewhere in the text.

**Table 4:** Descriptions of anti-adhesion assays applied to cranberry oligosaccharide fractions.

Bioassay Type (organisms/cells)	Assay Description	Active Fractions	Study
Bacterial HRBC Anti-Agglutination: *E. coli* (clinical strains)	Inhibition of the agglutination of HRBCs by *E. coli*. Results based on visual agglutination scores.	CJA, CJA1-02, CJA1-02B, CJA2-02, CJA2-03, CJA2-04, CJA2-05	[24,25,26,27,28]
Bacterial Anti-Adhesion: *E. coli* (CFT073) (radiolabeled) and T24 uroepithelial cells	Inhibition of the adhesion of *E. coli* to a confluent layer of epithelial cells. Results based on quantification of radioactivity	SNA, SNA-03, SNA-04	[27,28]
Bacterial Anti-Adhesion: *E. coli* (CFT073)(fluorescence labeled) and T24 uroepithelial cells	Inhibition of the adhesion of *E. coli* to a confluent layer of epithelial cells. Results based on quantification of fluorescence intensity	A2, A6, CCA, CCA1-01, CCA1-02, CCA1-03, CCA2-01, CCA2-02, CCA2-03, CCA2-04, CCA2-05,	[29,72,112]
Bacterial Anti-Adhesion: *E. coli* (UTI89) (fluorescence labeled) and T24 uroepithelial cells	Inhibition of the adhesion of *E. coli* to a confluent layer of epithelial cells. Results based on quantification of fluorescence intensity	A2, A6	[72,112]
Bacterial Anti-Adhesion: *E. coli* (O157:H7) HT29 colonic epithelial cells	Inhibition of the adhesion of *E. coli* to a confluent layer of epithelial cells. Results based on viable counts (CFU) of adhered bacteria after disruption of the cell layer.	A2, A6	[72,112]

HRBC = human red blood cells; CFU = colony forming units.

## Supplementary Materials

Excel spreadsheet/table containing descriptive details for each oligosaccharide fraction discussed in this manuscript and a list of all abbreviations used.
